# Impact of *Mlkl* or *Ripk3* deletion on age-associated liver inflammation, metabolic health, and lifespan

**DOI:** 10.1007/s11357-025-01553-5

**Published:** 2025-02-10

**Authors:** Sabira Mohammed, Phoebe Ohene-Marfo, Chao Jiang, Zongkai Peng, Nidheesh Thadathil, Albert Tran, Evan Nicklas, Shylesh Bhaskaran, Dawei Wang, Ramasamy Selvarani, Amit Singh, Zhibo Yang, Nagib Ahsan, Sathyaseelan S. Deepa

**Affiliations:** 1 Stephenson Cancer Center, Department of Biochemistry and Physiology, Oklahoma Center for Geroscience & Brain Aging, University of Oklahoma Health Sciences Center, 975 NE 10th Street, Oklahoma City, OK 73104 USA; 2 Department of Biochemistry & Physiology, University of Oklahoma Health Sciences Center, Oklahoma City, OK USA; 3 Department of Chemistry and Biochemistry, University of Oklahoma, Norman, OK USA; 4 Mass Spectrometry, Proteomics and Metabolomics Core Facility, Stephenson Life Sciences Research Center, The University of Oklahoma, Norman, OK USA; 5 Oklahoma Center for Geroscience & Brain Aging, University of Oklahoma Health Sciences Center, Oklahoma City, OK USA

**Keywords:** Inflammaging, Liver, Ripk3, Mlkl, Lifespan, MASLD

## Abstract

Chronic, low-grade inflammation is a hallmark of aging and various age-related diseases, including metabolic dysfunction-associated steatotic liver disease (MASLD). The prevalence of metabolic dysfunction-associated steatohepatitis (MASH), an advanced form of MASLD, increases with age and contributes to morbidity and mortality among the elderly. This study investigates the role of necroptosis, a programmed cell death pathway that promotes inflammation, in liver inflammaging and age-associated MASLD by utilizing genetic ablation models of two key necroptosis proteins, Mlkl or Ripk3. The absence of Mlkl or Ripk3 significantly reduced liver inflammation, steatosis, and fibrosis in aged male mice, supporting the role of necroptosis in age-associated MASLD. Additionally, Mlkl or Ripk3 deletion impacted other non-necroptotic cellular processes that drive inflammation and MASLD, such as cellular senescence, apoptosis, and autophagy in aged liver. Levels of plasma TNFα and IL6, key proinflammatory cytokines associated with inflammaging, are reduced in *Mlkl*^*−/−*^ or *Ripk3*^*−/−*^ aged mice, supporting a systemic effect of necroptosis inhibition on inflammation. Proteomic analysis of liver tissues emphasizes the critical role of lipid and immune regulatory processes in maintaining liver homeostasis when Mlkl or Ripk3 is absent in aging liver. While Mlkl deletion did not affect the lifespan of mice, Ripk3 deletion shortened it. Additionally, Mlkl deficiency improved insulin sensitivity, whereas Ripk3 deficiency exacerbated glucose intolerance in aged mice. Thus, selective inhibition of Mlkl, not Ripk3, represents a potential therapeutic avenue for mitigating age-related liver disease and enhancing metabolic outcomes in the elderly.

**Supplementary Information:**

The online version contains supplementary material available at 10.1007/s11357-025-01553-5.

## Introduction

Chronic, low-grade, sterile inflammation, termed “inflammaging,” is recognized as one of the “seven pillars of aging” [1, 2]. In humans, it is marked by increased circulating pro-inflammatory cytokines like IL-6, TNF-α, and IL-1β, which are linked to higher disease and mortality rates [3]. Age-related diseases such as cardiovascular disease, cancer, diabetes, and neurodegenerative disorders are associated with a persistent inflammatory state [4, 5]. Thus, chronic inflammation is a key factor in aging and age-related diseases [1].

Chronic inflammation in the aging liver, or liver inflammaging, is linked to metabolic dysfunction-associated steatotic liver disease (MASLD), a spectrum of liver disease conditions from simple steatosis to metabolic dysfunction-associated steatohepatitis (MASH), characterized by hepatocyte ballooning, inflammation, and fibrosis. MASLD/MASH prevalence nearly doubles in individuals aged 45–64 compared to those aged 20–44 and is associated with higher mortality in people aged 60–74 [6, 7]. MASH is a major risk factor for hepatocellular carcinoma (HCC) [8] and cardiovascular diseases [9] and is now the leading indication for liver transplantation in the elderly in the USA, with the proportion of elderly patients requiring transplants rising from 9% (2002–2005) to 23% (2018–2020) [10]. Despite many clinical trials, resmetirom remains the only FDA-approved drug for MASH (March 2024) [11], underscoring the need to better understand MASLD/MASH pathogenesis in aging.

Chronic inflammation is a key mechanism in MASLD development, making inflammaging a target for preventing age-related MASLD. Studies show that necroptosis, an inflammatory cell death pathway, contributes to liver inflammation and MASH in diet-induced MASLD/MASH mouse models [12, 13]. Necroptosis is triggered by stimuli (e.g., oxidative stress, TNFα), which sequentially activate receptor-interacting serine/threonine kinase 1 (Ripk1), Ripk3, and mixed lineage kinase domain-like (Mlkl) through phosphorylation, which then permeabilizes the membrane and releases DAMPs. DAMPs activate immune cells, increasing cytokine production and creating a feedback loop of inflammation and cell death [14]. Our group has shown that necroptosis markers increase with age in mouse livers, correlating with inflammation and MASH [15], and are elevated in the superoxide dismutase 1 knockout (*Sod1*^*−/−*^) mice, an accelerated aging model [16]. Treating aged WT or adult *Sod1*^*−/−*^ mice with necrostatin-1s, a RIPK1 inhibitor, reduces liver inflammation and MASH pathology, underscoring the necroptosis pathway’s role in age-related liver inflammation and MASH.

An increase in liver necroptosis markers correlates not only with liver inflammaging but also with lifespan in mice. While natural aging increases markers of necroptosis and inflammation in the livers of mice, these markers are significantly downregulated in the livers of aged Ames Dwarf mice, a mouse model of extended lifespan [17]. Conversely, markers of necroptosis and inflammation are increased in the livers of young *Sod1*^*−/−*^ mice that have reduced lifespan [16]. Therefore, to gain a better understanding of the role of necroptosis in liver inflammaging, age-associated MASLD, and lifespan, we genetically inhibited two key proteins in the necroptosis pathway, *Mlkl* or *Ripk3*, in mice. Our results show that the absence of *Mlkl* or *Ripk3* reduced liver inflammaging and age-associated MASLD pathology; however, the absence of these proteins had differential effects on lifespan and metabolic health. Additionally, the absence of *Mlkl* or *Ripk3* affected various cellular mechanisms involved in inflammation, including cellular senescence, autophagy, and apoptosis. These data indicate that Mlkl and Ripk3 have non-necroptotic roles, in addition to necroptosis, in regulating key pathways linked to inflammaging in the livers of aged mice.

## Methods

### Animals

All procedures were conducted according to the protocol approved by the Institutional Animal Care and Use Committee at the University of Oklahoma Health Sciences Center (OUHSC). *Ripk3*^*−/−*^ mice were obtained from Genentech (South San Francisco, CA, USA) [18], and *Mlkl*^*−/−*^ mice were provided by Dr. James Murphy (Walter and Eliza Hall Institute of Medical Research, Australia) [19]. We used *Ripk3*^*+/−*^ or *Mlkl*^*+/−*^ mice solely for breeding to generate *Ripk3*^+*/*+^, *Ripk3*^*−/−*^, *Mlkl*^+*/*+^, and *Mlkl*^*−/−*^ mice, and all mice were in C56BL/6 J background. Heterozygous mice produced during breeding were not included in the experiments. Mice were rehoused to form groups of wild-type and knockout mice from different litters. The mice were group housed in ventilated cages at 20 °C ± 2 °C and were fed with a normal chow diet (5053 Pico Lab, Purina Mills, Richmond, IN) on a 12-h dark/light cycle. For the lifespan study using male mice, we used 40 *Ripk3*^+*/*+^, 42 *Ripk3*^*−/−*^, 42 *Mlkl*^+*/*+^, and 45 *Mlkl*^*−/−*^ mice. Similarly, for the lifespan study using female mice, 45 *Ripk3*^+*/*+^, 44 *Ripk3*^*−/−*^, 44 *Mlkl*^+*/*+^, and 45 *Mlkl*^*−/−*^ mice were used. For survival analysis, the mice were allowed to live out their natural lifespan and the time of their spontaneous death and the ages were recorded. The mean, median, percentiles (10%, 25%, 75%, 90%), maximum lifespan for each group was calculated. For analyzing the glucose tolerance, insulin tolerance, and body composition, a separate cohort comprising of *n* = 10 mice/group was maintained. Body weight, lean mass, and fat mass were assessed every month by using the Quantitative Magnetic Resonance method (Bruker minispec LF90, MA, USA). For all other studies, we used liver tissue from mice that were generated and housed at the Oklahoma City Veterans Affairs Health Care System Animal Facility, and all procedures were approved by the Institutional Animal Care and Use Committee at the Oklahoma City Veterans Affairs Health Care System Animal Facility. For studies involving aged liver, we used young wild type (WT, 7 months) and old (24–25 months; WT, *Mlkl*^*−/−*^, and *Ripk3*^*−/−*^) male mice. The WT mice represent a mix of *Ripk3*^+*/*+^ and *Mlkl*^+*/*+^mice.

### Western blotting

Western blot analysis was performed as described previously [20]. The following primary antibodies were used: MLKL (Millipore Sigma, Burlington, MA); RIPK3 (Novus Biologicals, Centennial, CO); LC3I/II (Cell Signaling Technology, Danvers, MA); β-actin (Sigma-Aldrich, St. Louis, MO). HRP-linked secondary antibodies were from Cell Signaling Technology. Images were taken with the Chemidoc imager (Bio-Rad) and quantified with ImageJ software (US National Institutes of Health).

### Immunohistochemistry (IHC) staining

IHC staining was performed using paraffin-embedded liver sections for P-MLKL (Abcam, Cambridge, UK); F4/80 (Proteintech, Rosemont, IL); and Cleaved Caspase 3 (Cell Signaling Technology) using a standardized protocol [21]. Images were taken using an ECHO REVOLVE R4 microscope for three random non-overlapping fields per sample. For P-MLKL, staining intensity was quantified using ImageJ software by using the color deconvolution plug-in, and the percentage area of DAB (3, 3′-diaminobenzidine) staining was obtained for quantification. For F4/80 and Cleaved Caspase 3 staining, the number of positively stained cells per field was quantified using the Cell Count feature in the Echo Revolve R4 microscope. Three random fields per sample were acquired.

### Quantitative real-time PCR (RT-PCR)

RNA was isolated from 20-mg frozen liver tissues, and the real-time-PCR was performed as described previously [20]. The calculations were performed by a comparative method (2^−ΔΔCt)^ using β-microglobulin, β-actin, or hypoxanthine phosphoribosyltransferase 1 (HPRT) as housekeeping genes. The data are represented as fold change after normalization to the young WT mice group. The primers used are listed in Table S2.

### Picrosirius red (PSR) staining

PSR staining was done using a standard protocol [21]. The images were taken using an ECHO REVOLVE R4 microscope for 3 random non-overlapping fields per sample. The percentage area of PSR staining was quantified using the thresholding option in ImageJ software.

### Histological analysis

Formalin-fixed liver tissue was embedded in paraffin and sectioned. The sections were then stained with Hematoxylin & Eosin (H&E) using a standard protocol [21]. Images were taken using an ECHO REVOLVE R4 microscope for 3 random non-overlapping fields per sample. The hepatic steatosis was quantified using the Cell Count option in Echo Revolve R4 microscope and is represented graphically.

### Quantification of liver triglyceride

Liver triglyceride levels were quantified using a triglyceride colorimetric assay kit from Cayman Chemical Company (Ann Arbor, MI, USA) as described [20], following manufacturer’s instructions.

### Glucose tolerance (GTT) and insulin tolerance tests (ITT)

GTT and ITT were performed as described before [20]. Briefly, mice were fasted for 6 h for GTT and received intraperitoneal injection of glucose (2 g/kg, Sigma-Aldrich) or 5 h for ITT and received insulin (0.75 units/kg, Novo Nordisk Inc., Bagsvaerd, Denmark). Blood glucose concentration was measured before glucose or insulin injection and then 15, 30, 60, and 120 min after administration using TRUE METRIX glucose strips and glucometer (Trividia Health Inc., Plainsboro Township, NJ, USA).

### Plasma analyses for high-mobility group box-1(HMGB1), alanine transaminase (ALT), and pro-inflammatory cytokines

The levels of ALT and HMGB1 in plasma were measured using ALT colorimetric activity assay kit from Cayman Chemical Company and mouse HMGB1 ELISA Kit (Elabscience, Houston, TX) as per manufacturer’s instructions. The pro-inflammatory cytokines in plasma were determined using Meso Scale Discovery V-PLEX Custom Mouse Biomarkers Proinflammatory Panel1 (K152A0H-1, MSD, Rockville, MD).

### Proteomic analysis of liver samples

A total of 100 µg of liver proteins (*n* = 5/group) were subjected for in-solution trypsin/LysC (Cat# V5071, Promega, WI, USA) digestion. Trypsin digestion was performed according to the manufacturer’s protocol. Following digestion, the peptides were desalted using C18 Sep-Pak Plus cartridges (Waters, MA, USA). The dried tryptic peptides were reconstituted with 100 µL of 0.1% formic acid to a final concentration of 1 µg/µL. The resuspended tryptic peptides (2 μL) were loaded onto a C18 trap column (150 μm × 3 cm, 3 μm resin, Acclaim™ PepMap™ 100 C18 HPLC Column, Thermo Scientific™, USA) using mobile phase A (0.1% formic acid in LC–MS grade water) at a flow rate of 3 μl/min for 10 min, and separate peptides on an EASY-Spray™ HPLC analytical column (3 μm × 75 μm × 15 cm, Catalog # ES900 Thermo Scientific™, USA) at 350 nL/min. The total LC–MS/MS run time is 60 min, including column wash and re-equilibration. The LC–MS/MS analysis was conducted using a Dionex UltiMate® 3000 UHPLC system (Thermo Fisher Scientific, CA, USA) coupled to a Q Exactive HF-X mass spectrometer (Thermo Fisher Scientific, Waltham, MA) as described previously [22].

The RAW MS files were searched against the UniProt reviewed mouse (Taxon ID: 10,090) protein database using the Sequest algorithm within Proteome Discoverer v 2.4 (Thermo Fisher Scientific, San Jose, CA). Parameters used for the Sequest database search are listed as follows: trypsin enzyme cleavage specificity, 2 possible missed cleavages, 10 ppm mass tolerance for precursor ions, and 0.02 Da mass tolerance for fragment ions. Search parameters permit dynamic modification of methionine oxidation (+ 15.9949 Da) and static modification of carbamidomethylation (+ 57.0215 Da) on cysteine. Peptide assignments from the database search are filtered down to a 1% FDR (false discovery rate). Label-free quantitation across the samples employs the Minora algorithm and the adjoining bioinformatics tools available in Proteome Discoverer. A 1.5-fold increase or decrease in abundance with a *p*-value < 0.05 is considered statistically significant. Proteomics data can be found in the MassIVE database via MSV000096299.

### Bioinformatics

Heatmaps and volcano plots were generated by SRplot (https://www.bioinformatics.com.cn/en), a free online platform for data analysis and visualization. Venn diagram was generated using an open-source platform (https://pnnl-comp-mass-spec.github.io/Venn-Diagram-Plotter/). All pathway analyses were performed using ShinyGO 0.80 bioinformatics platform [23]. The remaining parts were generated by Microsoft Office PowerPoints and Excel 365.

### Statistical analysis

All data are represented as mean ± SEM. Ordinary one-way ANOVA with uncorrected Fisher’s LSD test was used to analyze data with GraphPad Prism. *F*-values and *p*-values from the ANOVA summary are included in the figure legends to provide a complete representation of the statistical analysis. For survival curve analysis, the mean, median, and percentile values were obtained by performing descriptive statistics analysis for the data simple survival analysis (Kaplan–Meier) using GraphPad Prism. Mann–Whitney test was performed on the data for statistical significance. The significance between the survival curves was analyzed by performing Mantel-Cox test followed by Gehan-Breslow-Wilcoxon test. *p* < 0.05 is considered statistically significant.

## Results

### Absence of *Mlkl *or *Ripk3* reduced liver inflammation in aged mice

**Fig. 1:**
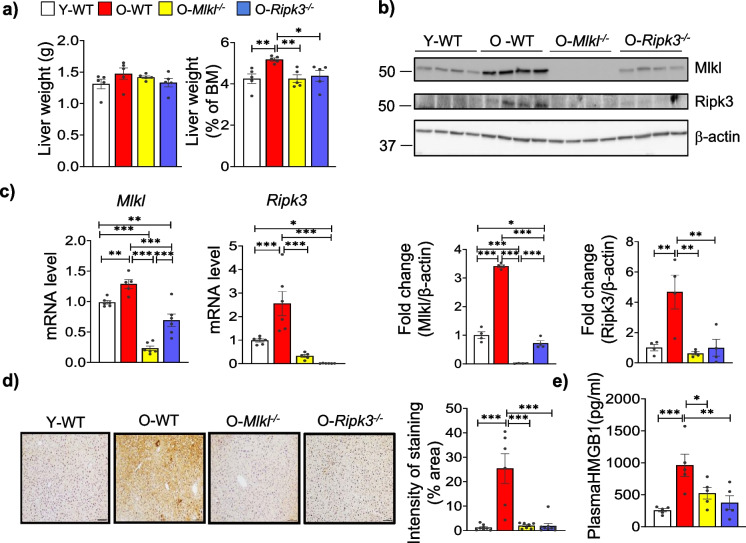
Markers of necroptosis in young and old livers. **a** Gross liver weight in grams (on the left) and percentage liver weight (normalized to body mass (BM) (on the right) of young (7-month-old) WT or old WT, *Mlkl*^*−/−*^ or *Ripk3*^*−/−*^ (24-month-old) male mice. **b** Top: Immunoblots of liver tissue extracts for necroptosis proteins: Mlkl, Ripk3 and β-actin. Bottom: Graphical representation of quantified blot normalized to β-actin. **c** Transcript levels of *Mlkl* and *Ripk3*. **d** *Left*: Representative IHC staining for P-MLKL in liver sections. *Right*: Graphical representation of the intensity of staining. Scale bar: 50 µm. **e** Levels of HMGB1 in circulation. White, red, yellow, and blue bars represent young WT, old WT, old *Mlkl*^*−/−*^, and old *Ripk3*.^*−/−*^, respectively (*n* = 4–6 groups). Error bars are represented as mean ± SEM. One-way ANOVA, *p < 0.05, **p < 0.005, ***p < 0.0005. ANOVA summary (*F* value, *p*-value): (**a**, left) 1.2, 0.34; (**a**, right) 4.83, 0.01; (**b**, left) 364.6, < 0.0001; (**b**, right) 9.04, 0.002; (**c**, left) 43.6, < 0.0001; (**c**, right) 17.88, < 0.0001; (**d**) 19.37, < 0.0001; (**e**) 7.48, 0.002

To evaluate the impact of *Mlkl* or *Ripk3* deficiency on age-associated MASLD, we used *Mlkl*^*−/−*^ or *Ripk3*^*−/−*^ male mice. A combination of *Mlkl*^+*/*+^ or *Ripk3*^+*/*+^ aged mice were used as old wild-type (WT) controls, as our data show that the body weight and expression of *Mlkl* or *Ripk3* are similar in both *Mlkl*^+*/*+^ and *Ripk3*^+*/*+^ mice (Fig S1a, b). The liver weight remained unchanged between the study groups (Fig. 1a). However, the liver weight, when normalized to body weight, was significantly higher in old WT mice compared to young mice, whereas the absence of *Mlkl* or* Ripk3* significantly reduced liver weight in aged mice (Fig. 1a). Protein expression and transcript levels of Mlkl (3.4-fold and 1.3-fold) and Ripk3 (4.6-fold and 2.5-fold) were significantly elevated in the livers of old WT mice, compared to young WT mice (Fig. 1b–c). The absence of *Mlkl* reduced *Ripk3* expression, and vice versa, at both transcript and protein levels (Fig. 1b–c). Measurement of phosphorylated Mlkl (P-Mlkl), a marker of necroptosis, by immunostaining of liver tissues showed a 20-fold increase in P-MLKL staining in the livers of old WT mice compared to young WT mice, while both *Mlkl*^*−/−*^ and *Ripk3*^*−/−*^ old mice showed significant reduction in P-Mlkl staining (Fig. 1d). The circulating level of HMGB1, a pro-inflammatory DAMP released during necroptosis, was significantly increased in the plasma of old WT mice and was reduced in both aged knockout mice (Fig. 1e).

**Fig. 2:**
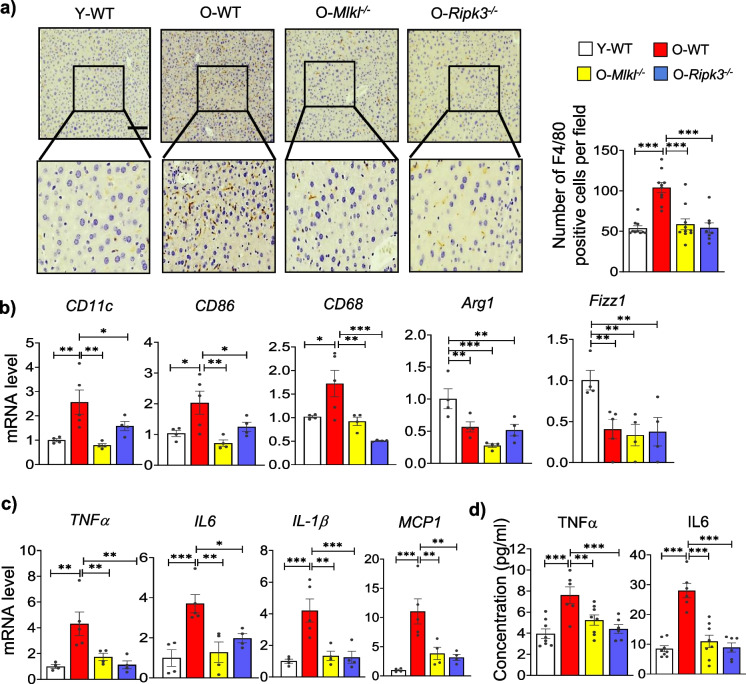
Absence of *Mlkl* and *Ripk3* reduces hepatic inflammation. **a** *Left*: Representative images for IHC staining for F4/80 (brown) counterstained with hematoxylin (purple) in liver sections of experimental mice. *Right*: Graphical representation of number of F4/80 positive cells detected per microscopic field. Scale bar: 100 µm. Transcript levels (**b**) *CD11c*, *CD86*, and *CD 68*, *Arg1* and *Fizz1* (**c**) *TNFα*, *IL6*, *IL-1β* and *MCP1* normalized with respect to housekeeping genes and represented as fold change relative to young WT group. (**d**) Circulating levels of TNFα, and IL6 (*n* = 6–8/groups). White, red, yellow, and blue bars represent young WT, old WT, old *Mlkl*^*−/−*^, and old *Ripk3*.^*−/−*^, respectively (Figures **a**–**c**, *n* = 4–6 group). Error bars are represented as mean ± SEM. One-way ANOVA, *p < 0.05, **p < 0.005, ***p < 0.0005. ANOVA summary (*F* value, *p-*value): (**a**) 18.3, < 0.0001; (**b**, *CD11c*) 6.85, 0.005; (**b**, *CD86*) 5.6, 0.01; (**b**, *CD68*) 8.8, 0.002; (**b**, *Arg1*) 9.45, 0.002; (**b**, *Fizz1*) 5.25, 0.014; (**c**, *TNFα*) 7.73, 0.003; (**c**, *IL6*) 8.95, 0.002; (**c**, *IL-1β*) 10.2, 0.001; (**c**, *MCP1*) 10.5, 0.0009; (**d**, *TNFα*) 8.42, 0.0005; (**d**, IL6) 24.26, < 0.0001

Immunostaining for the macrophage marker, F4/80, showed that aging resulted in a significant increase in the number of F4/80 positive cells in the liver (1.9-fold) (Fig. 2a). Additionally, markers of proinflammatory macrophages CD11c, CD86, and CD68 (2.6-fold, 1.9-fold, 1.7-fold) were significantly upregulated, and markers of anti-inflammatory macrophages Arg1 (0.6-fold) and Fizz1 (0.4-fold) were significantly downregulated in old WT mice relative to young mice (Fig. 2b). The absence of *Mlkl* or *Ripk3* significantly reduced the number of F4/80 positive cells and proinflammatory macrophage markers in aged mice; however, anti-inflammatory macrophage markers were unaffected by the absence of either *Mlkl* or *Ripk3*. Consistent with the increased levels of proinflammatory macrophage markers, the transcript levels of pro-inflammatory cytokines associated with inflammaging (TNFα, IL6, and IL1β) and the chemokine MCP1 were significantly upregulated in the livers of old WT mice compared to young mice (TNFα, 4.3-fold; IL6, 3.7-fold; IL1β, 4.4-fold; MCP1, 11.8-fold), and the absence of *Mlkl* or *Ripk3* significantly reduced their levels in aged mice (Fig. 2c). The levels of circulating TNFα (1.9-fold) and IL6 (3.3-fold) were significantly elevated in old WT mice relative to young mice, while *Mlkl*^*−/−*^ or *Ripk3*^*−/−*^ old mice showed a significant reduction in their levels compared to aged WT mice (Fig. 2d). Thus, our data show that the absence of *Mlkl* or *Ripk3* mitigates age-related proinflammatory macrophage accumulation and hepatic inflammation.

### Aged *Mlkl*^−/−^ or *Ripk3*^−/−^ mice exhibited reduced steatosis and liver fibrosis

**Fig. 3:**
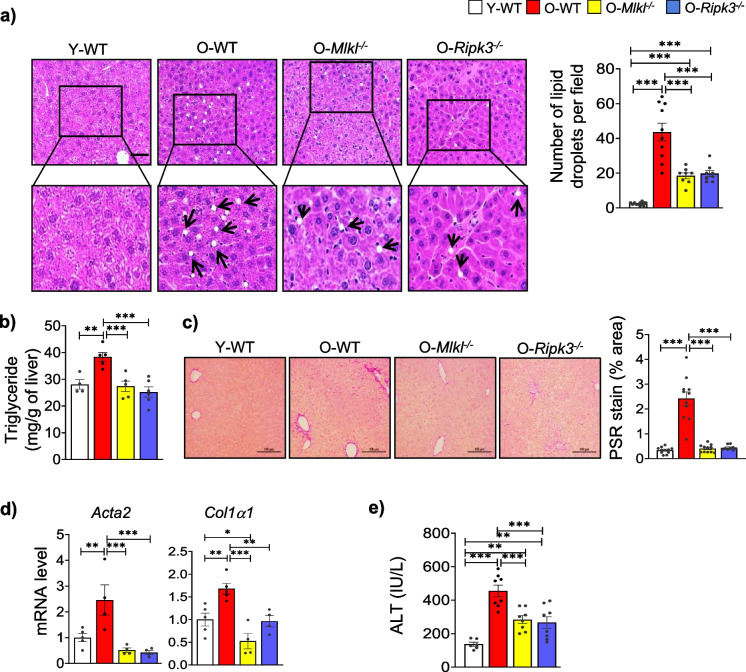
Absence of *Mlkl* and *Ripk3* protects from age related liver pathology. **a** *Left*: Representative images of H&E-stained liver sections. Scale bar: 100 µM; *Right*: Graphical representation of steatosis in the livers of experimental mice groups. **b** Quantification of total triglyceride in liver tissue. **c** *Left:* Representative images of PSR staining of liver sections. Scale bar: 100µM. *Right:* Quantification of PSR staining, represented as percentage area. **d** The transcript levels of fibrosis markers normalized with respect to housekeeping genes and represented as fold change relative to young WT group. **e** Levels of ALT in plasma (*n* = 6–8 group). White, red, yellow, and blue bars represent young WT, old WT, old *Mlkl*^*−/−*^, or old *Ripk3*.^*−/−*^, respectively (Figures **a**–d: *n* = 4–6 groups). Error bars are represented as mean ± SEM. One-way ANOVA, *p < 0.05, **p < 0.005, ***p < 0.0005. ANOVA summary (*F* value, *p*-value): (**a**) 39.74, < 0.0001; (**b**) 10.14, 0.0006; (**c**) 50.6, < 0.0001; (**d**, *Acta2*) 9.59, 0.001; (**d**, *Col1α1*) 11.97, 0.0004; (**e**) 18.66, < 0.0001

H&E staining of liver tissues showed that aging-induced microvesicular steatosis was significantly reduced in both *Mlkl*^*−/−*^ and *Ripk3*^*−/−*^ old mice (Fig. 3a). In line with this, hepatic triglyceride content was 1.4-fold higher in old WT mice compared to young mice, while the absence of *Mlkl* or *Ripk3* significantly reduced triglyceride levels in aged mice (Fig. 3b). Assessment of liver fibrosis using PSR staining that detects collagen fibers in a tissue revealed a significant increase in PSR staining in old WT mice (7.3-fold) compared to young mice, whereas *Mlkl*^*−/−*^ or *Ripk3*^*−/−*^ aged mice showed a significant reduction in PSR staining (Fig. 3c). This observation was consistent with transcript levels of fibrosis markers, which were significantly elevated in old WT mice (Acta2, 2.2-fold; Col1α1, 1.7-fold) compared to young mice, while the absence of *Mlkl* or *Ripk3* significantly reduced their levels in aged mice (Fig. 3d). Additionally, plasma ALT level, an indicator of liver injury, increased by nearly 3.3-fold in old WT mice but was significantly reduced in the absence of *Mlkl* or *Ripk3* (Fig. 3e). Thus, the absence of *Mlkl* or *Ripk3* protects mice against age-related MASLD/MASH pathology by reducing liver steatosis, fibrosis, and liver injury.

### Absence of *Mlkl* or *Ripk3* reduced cellular senescence markers in aged liver

**Fig. 4:**
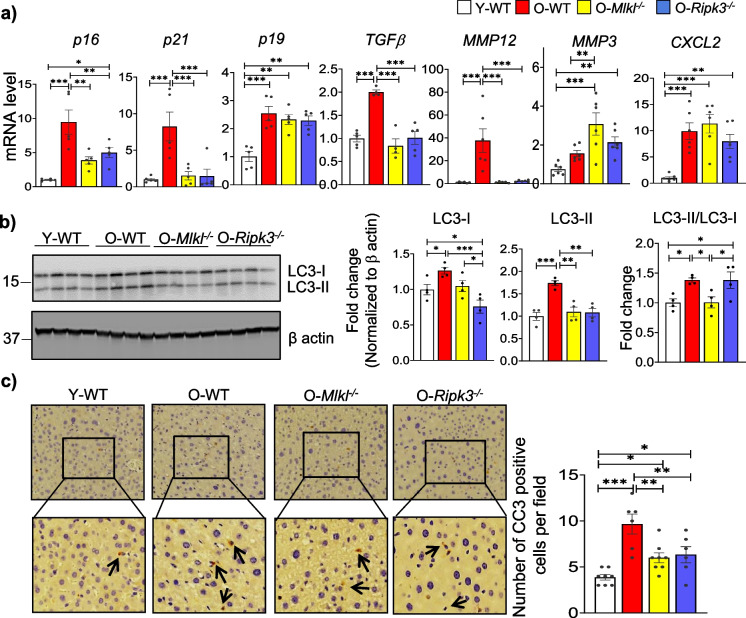
Effect of absence of *Mlkl* and *Ripk3* on non-canonical functions in aged liver. **a** The transcript levels of *p16*, *p21*, *p19*, *TGFβ*, *MMP12*, *MMP3*, and *CXCL2* normalized with respect to housekeeping genes and represented as fold change relative to young WT group. **b** *Left*: Immunoblots of liver tissue extracts for LC3-I, LC3-II (autophagy markers) and β-actin. *Right*: Graphical representation of quantified immunoblot normalized to β-actin and ratio of LC3-II/I. *Left:* Representative images for IHC staining for cleaved caspase-3 in liver sections. *Right:* Graphical representation of number of cleaved caspase-3 positive cells (arrow heads) per microscopic field. Scale bar: 100 µm. White, red, yellow, and blue bars represent young WT, old WT, old *Mlkl*^*−/−*^, or old *Ripk3*.^*−/−*^ respectively (*n* = 4–6/groups). Error bars are represented as mean ± SEM. One-way ANOVA, *p < 0.05, **p < 0.005, ***p < 0.0005. ANOVA summary (*F* value, *p*-value): (**a**, *p16*) 12.49, 0.0002; (**a**, *p21*) 9.34, 0.0008; (**a**, *p19*) 12.59, 0.0002; (**a**, *TGFβ*) 20.46, < 0.0001; (**a**, *MMP12*) 11.57, 0.0002; (**a**, MMP3) 8.62, 0.0007; (**a**, CXCL2) 11.15, 0.0002; (**b**, LC3-I) 7.88, 0.0036; (**b**, LC3-II) 12.19, 0.0006; (**b**, LC3-II/LC3-I) 5.73, 0.01; (**c**) 11.64, < 0.0001

Our previous study has shown that pharmacological inhibition of necroptosis with necrostatin-1s reduced markers of cellular senescence in the livers of old WT mice [15]. Based on these findings, we investigated the effect of genetic inhibition of necroptosis pathway proteins on cellular senescence in aged liver. Markers of cellular senescence (p16, 9.5-fold; p21, 8.2-fold; p19, 2.5-fold) and senescence associated secreted factors, SASP (TGFβ,  2- fold; MMP12, 40-fold; CXCL2, 10-fold) were significantly upregulated in the livers of old WT mice compared to young mice, except for MMP3 (Fig. 4a). The absence of *Mlkl* or *Ripk3* significantly reduced the levels of p16, p21, TGFβ, and MMP12 in aged livers, while levels of other genes remained unaffected by the lack of *Mlkl* or *Ripk3* (Fig. 4a).

### Absence of *Mlkl* or *Ripk3* impacted non-necroptotic functions of these proteins in aged livers

Both Mlkl and Ripk3 have several non-canonical functions that are independent of necroptosis. Wu et al. reported that a western diet that promotes MASH increases the expression of autophagy marker microtubule-associated protein 1 light chain 3-II (LC3-II) in the liver of mice, indicating reduced autophagic flux, and deficiency of *Mlkl* blocked this effect of western diet [24]. Therefore, we measured expressions of LC3-I and LC3-II in aged liver. Protein expression of LC3-II (1.7-fold) and its precursor LC3-I (1.26-fold) were significantly upregulated in the livers of old WT mice compared to young mice, and the absence of *Ripk3*, not *Mlkl*, significantly reduced their expression (Fig. 4b). Analysis of the LC3-II/LC3-I ratio revealed a significant upregulation in old WT mice (1.4-fold) compared to young mice, which was reduced in the absence of *Mlkl*, but not *Ripk3* (Fig. 4b).

Increased apoptosis of hepatocytes promotes MASH [25, 26], and Ripk3 promotes apoptosis independent of its role in necroptosis [27]. Therefore, we measured the expression of cleaved caspase-3 (CC3) in the liver, an apoptosis marker, via immunohistochemical staining. There was a 2.5-fold increase in the number of CC3-positive cells in the liver of old WT mice compared to young mice, and *Mlkl*^*−/−*^ or *Ripk3*^*−/−*^ old mice showed a significant reduction in liver CC3 staining (Fig. 4c).

### Impact of *Mlkl* or *Ripk3* deficiency on the liver proteome in aging

**Fig. 5:**
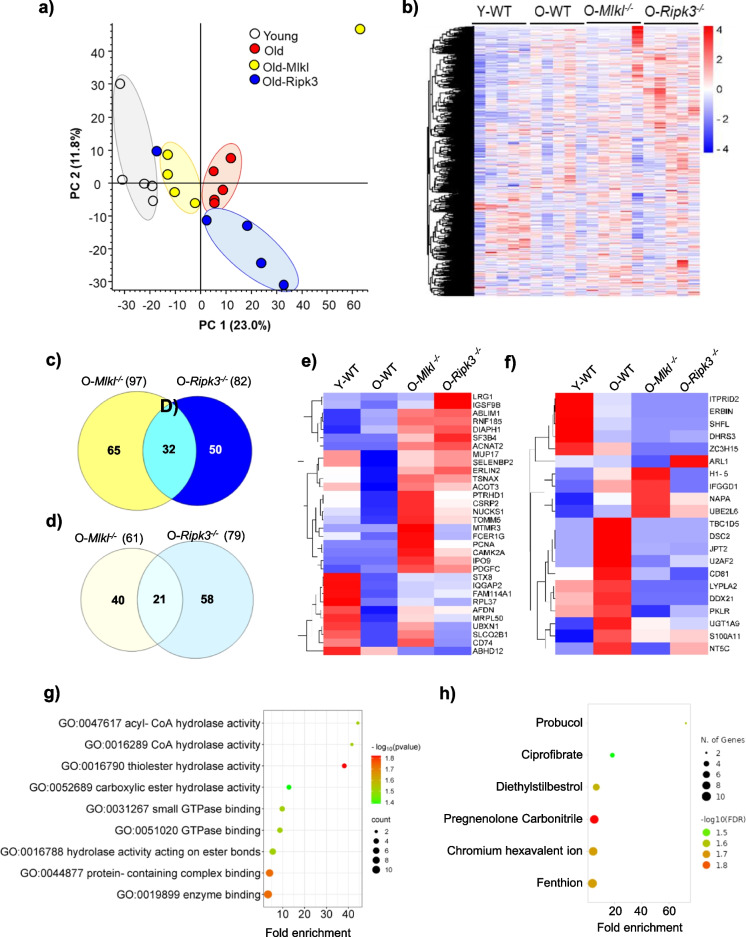
Effect of absence of *Mlkl* and *Ripk3* on aging liver proteome. **a** Principal component analysis (PCA) score plot of proteomics data from livers of young WT (white circles), old WT (red circles), old *Mlkl*^*−/−*^ (yellow circles), or old *Ripk3*^*−/−*^ (blue circles) mice. (*n* = 5 per group). **b** Heat map clustering of the protein abundance of the total unique identified proteins in the experimental groups. Venn diagram of the number of commonly upregulated (**c**) and downregulated (**d**) proteins in the livers of old *Mlkl*^*−/−*^ and old *Ripk3*^*−/−*^ mice. Heat map analyses of the commonly upregulated (**e**) and downregulated (**f**) proteins in the livers of old *Mlkl*^*−/−*^ and old *Ripk3*.^*−/−*^ mice. **g** Gene ontology (GO) molecular pathway analysis of the commonly upregulated proteins in (**c**) and (**e**). **h** Drug comparative toxicogenomics database (Drug.CTD) enrichment bubble analysis of the commonly upregulated proteins in (**c**) and (**e**)

A label-free quantitative proteomic analysis of liver tissues from young WT, old WT, *Mlkl*^*−/−*^, and *Ripk3*^*−/−*^ mice successfully identified and quantified a total of 2614 unique protein groups (Table S1). A principal component analysis (PCA) of total protein abundance revealed that, despite their differences, the biological replicates in each group tightly clustered together, showing the heterogeneous nature of the liver samples (Fig. 5a). Similarly, heat map clustering of the protein abundance of the total unique identified proteins further demonstrates distinct cluster among the groups (Fig. 5b). Volcano plot analysis further revealed the significant (at least 1.5-fold up or down with an adjusted *p*-value < 0.05) difference of several proteins when compared between the groups (Figure S2a–b). A total of 97 proteins were significantly increased, whereas 61 proteins were significantly decreased in abundance in *Mlkl*^*−/−*^ mice compared to the aged WT mice (Figure S2a). Similarly, a total of 82 proteins were significantly increased, while 79 proteins were significantly decreased in abundance in *Ripk3*^*−/−*^ mice compared to the old WT mice (Figure S2b). A comparison of the up- and down-regulated proteins in liver samples from the aged *Mlkl*^*−/−*^ and *Ripk3*^*−/−*^ with those from the aged WT mice revealed that several proteins were commonly altered between the groups (Fig. 5c–d). Heat map analyses of the commonly upregulated and downregulated proteins in both *Mlkl*^*−/−*^ and *Ripk3*^*−/−*^ mice, relative to aged WT mice, are shown in Fig. 5e and f. The full list of these proteins is provided in Table S1.

The dot plot in Fig. 5g shows enriched Gene Ontology (GO) terms for molecular functions in the dataset. Pathway enrichment analysis revealed that the acyl-CoA hydrolase activity has the highest fold enrichment, whereas the thioester hydrolase activity pathway showed the highest statistical significance (Fig. 5g). Overall, a strong enrichment of proteins involved in lipid metabolism, broad hydrolase activity, GTPase binding, and protein complex interactions were commonly upregulated in both *Mlkl*^*−/−*^ and *Ripk3*^*−/−*^ mice livers (Table S1). Proteins such as Acot3 and Acnat2 are associated with hydrolase activities related to acyl-CoA and CoA, suggesting a role in lipid metabolism [28]. Additionally, proteins including Acot3, Abhd12, Acnat2, and Ptrhd1 exhibit various hydrolase activities acting on ester bonds, carboxylic esters, and thiol esters, suggesting adaptive shifts in metabolic processes when necroptotic pathways are disrupted [29–32]. The presence of Diaph1, Iqgap2, Ipo9, and Afdn linked to GTPase and small GTPase binding highlights their involvement in cellular signaling and cytoskeletal organization, likely compensating for structural changes and communication needs due to necroptosis inhibition [33–36]. Furthermore, proteins such as Iqgap2, Afdn, Rnf185 and Cd74 show associations with protein complex and enzyme binding, indicating potential roles in regulating cellular processes, immune responses, and stress adaptations [37–40]. Additionally, a strong correlation exists between proteins commonly upregulated in *Mlkl*^*−/−*^ and *Ripk3*^*−/−*^ mice livers and the drug pregnenolone carbonitrile (Fig. 5h).

### Absence of Mlkl did not impact lifespan whereas absence of Ripk3 reduced lifespan

**Fig. 6:**
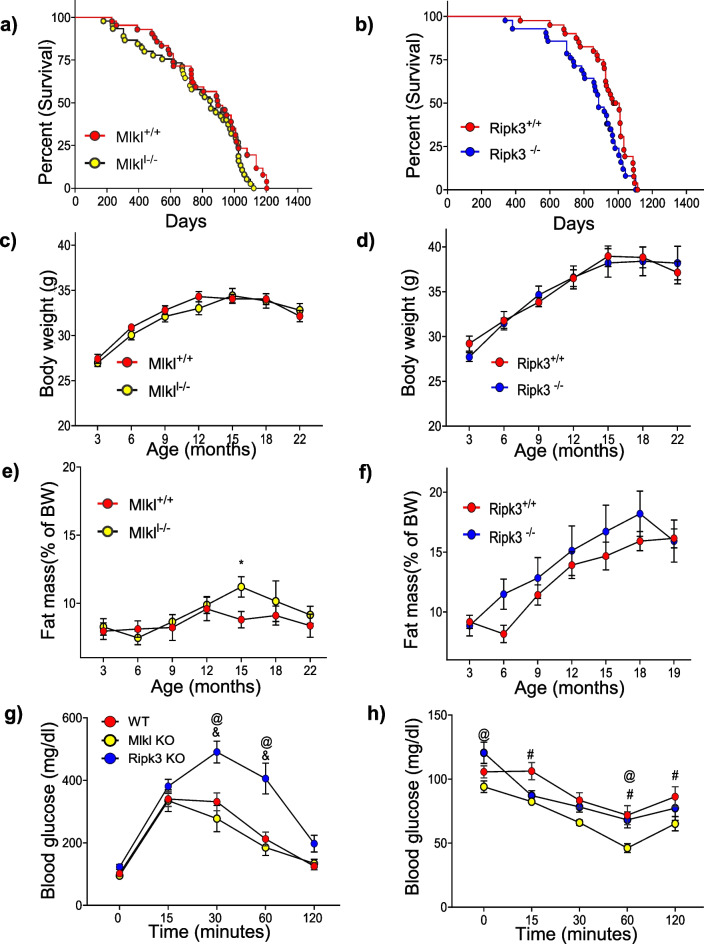
Effect of absence of *Ripk3 or Mlkl* on survival, body weight and body composition of male mice. Kaplan–Meier survival curves for *Mlkl*^+*/*+^, *Mlkl*^*−/−*^ (on the left) mice (**a**) or *Ripk3*^+*/*+^, *Ripk3*^*−/−*^ mice (on the right) (**b**). Average body weight changes of *Mlkl*^+*/*+^, *Mlkl*^*−/−*^ (**c**) and *Ripk3*^+*/*+^*, Ripk3*^*−/−*^ (**d**) mice. Percentage of fat mass normalized to total body weight of *Mlkl*^+*/*+^, *Mlkl*^*−/−*^ (**e**) and *Ripk3*^+*/*+^*, Ripk3*^*−/−*^ (**f**) mice. Glucose tolerance test (GTT) (**g**) and Insulin tolerance test (ITT) (**h**) of old WT, old *Mlkl*^*−/−*^, and old *Ripk3*^*−/−*^ mice (all three groups were 22 months of age). GTT and ITT were performed with the same mice, with a time gap of 10 days between the tests. **a**, **b**: *n* = 42 *Mlkl*^+*/*+^, *n* = 45 *Mlkl*^*−/−*^, *n* = 40 *Ripk3*^+*/*+^, *n* = 42 *Ripk3*^*−/−*^. **c**–**h**: *n* = 10/ group. Data are expressed as mean ± SEM and analyzed using two-way ANOVA. *p < 0.05. For **g**, **h**: #: represents significant difference between old WT vs old *Mlkl*^*−/−*^, &: old WT vs old *Ripk3*^*−/−*^, @: old *Mlkl*^*−/−*^ vs old *Ripk3*.^*−/−*^. ANOVA summary (*F* value, *p*-value): (**c**) row factor (time points) 36.24, < 0.0001 column factor (genotype) 1.28, 0.26; (**d**) row factor (time points) 22.56, < 0.0001 column factor (genotype) 0.052, 0.82; (**e**) row factor (time points) 2.61, 0.021 column factor (genotype) 2.64, 0.107; (**f**) row factor (time points) 11.08, < 0.0001 column factor (genotype) 3.73, 0.056; (**g**) row factor (time points) 57.49, < 0.0001 column factor (genotype) 26.55, < 0.0001; (**h**) row factor (time points) 22.48, < 0.0001 column factor (genotype) 12.74, < 0.0001

To determine the effect of *Mlkl or Ripk3* deficiency on overall survival, we examined the lifespan of *Mlkl*^+*/*+^, *Mlkl*^*−/−*^, *Ripk3*^+*/*+^, and *Ripk3*^*−/−*^ male mice. Table 1 provides a detailed analysis of lifespan data, including mean (the average age at death for the study population), median (the age by which 50% of the population has died), and maximal lifespan (the age at death of the longest-lived individual). Mean, median, and maximum lifespan did not differ significantly between *Mlkl*^+*/*+^ and *Mlkl*^*−/−*^ male mice (Fig. 6a, Table 1). However, mean and median lifespan of *Ripk3*^*−/−*^ male mice was significantly lower than *Ripk3*^+*/*+^ male mice, with no difference in their maximal lifespan (Fig. 6b, Table 1). Similar results were observed for *Mlkl*^*−/−*^ or *Ripk3*^*−/−*^ female mice (Figure S3a–b, Table 1). Body composition analysis throughout the lifespan revealed no significant differences in body weight for male or female *Mlkl*^+*/*+^ or *Mlkl*^*−/−*^ mice and *Ripk3*^+*/*+^ or *Ripk3*^*−/−*^ (Fig. 6c–d, S3c–d). Similarly, there was no significant difference in fat mass, percentage fat mass, lean mass, and percentage lean mass for male or female *Mlkl*^+*/*+^ or *Mlkl*^*−/−*^ mice and *Ripk3*^+*/*+^ or *Ripk3*^*−/−*^ mice at 22-months of age (Fig. 6e–f, S4a–d, S5a–c).

**Table 1:** Lifespan analysis of male and female *Mlkl*^+*/*+^ and *Mlkl*^*−/−*^ and *Ripk3*^+*/*+^ and *Ripk3*^*−/−*^ mice

Males		
	***Mlkl***^**+*/*+**^	***Mlkl***^***−/−***^
Mean ± SEM	806.6 ± 38^@^	769.2 ± 41.32
Median	847.5	853.0
Maximum	1204	1122
10% Percentile	488.1	304.0
25% Percentile	616.0	584.5
75% Percentile	981.3	1016
90% percentile	1137	1054
Number of mice	42	45
	***Ripk3***^**+*/*+**^	***Ripk3***^***−/−***^
Mean ± SEM	930.45 ± 22.65*	838.6 ± 28.74
Median	976.5*	885
Maximum	1114	1103
10% Percentile	695.5	578.2
25% Percentile	889.5	734.3
75% Percentile	1014	959.5
90% percentile	1090	1024
Number of mice	40	42
Females		
	***Mlkl***^**+*/*+**^	***Mlkl***^***−/−***^
Mean ± SEM	786.4 ± 27.73	834.9 ± 26.13^#^
Median	839	911
Maximum	1068	1056
10% Percentile	448.5	536.6
25% Percentile	728.8	729.5
75% Percentile	907.0	957.5
90% percentile	998.5	990.8
Number of mice	44	45
	***Ripk3***^**+*/*+**^	***Ripk3***^***−/−***^
Mean ± SEM	839 ± 26.84*	742.8 ± 34.11
Median	889.0	872.0
Maximum	1104	1029
10% Percentile	521.4	351.5
25% Percentile	793.5	562.5
75% Percentile	949.0	914.5
90% percentile	1004	962.5
Number of mice	45	44

The survival data from Fig. 6 and Figure S2 are expressed in days. “*” indicates significant difference between genotypes of same sex. “@” indicates significant difference between male *Mlkl*^+*/*+^ and *Ripk3*^+*/*+^ mice. “#” indicates significant difference between female *Mlkl*^*−/−*^ and *Ripk3*^*−/−*^ mice

Next, we performed GTT and ITT in male mice to evaluate the effects of *Mlkl* or *Ripk3* deficiency on metabolic health in aged mice. The results indicated that aged WT and *Mlkl*^*−/−*^ mice had similar glucose tolerance. However, aged *Ripk3*^*−/−*^ mice displayed glucose intolerance (Fig. 6g, S5d). In contrast, the ITT results showed that insulin sensitivity was comparable between WT and *Ripk3*^*−/−*^ old mice, while aged *Mlkl*^*−/−*^ mice exhibited improved insulin sensitivity (Fig. 6h, S5e). These data suggest that *Mlkl* or *Ripk3* deficiency exerts distinct effects on lifespan and metabolic health in aged male mice.

## Discussion

The aim of our study was to determine the effect of absence of Mlkl or Ripk3, two key necroptosis pathway proteins, on age-related liver pathology, lifespan, and metabolic health in naturally aged mice. In our study, we targeted both Mlkl and Ripk3 due to reports of several non-canonical functions associated with these proteins [41, 42]. Therefore, if deleting Mlkl or Ripk3 has similar effects on outcome measures, we will be able to establish with relative certainty that necroptosis plays a role in driving the observed pathological processes. Our findings reveal that the genetic deletion of *Mlkl* or *Ripk3* protects mice from age-related hepatic inflammation, steatosis, and fibrosis, hallmarks of MASLD. Notably, the absence of *Mlkl* or *Ripk3* differently affected lifespan and metabolic health in aged mice.

Consistent with our previous report [15], we found that aging increases MLKL phosphorylation (P-MLKL), a necroptosis marker, in aged livers. An increase in necroptosis markers is reported in mouse models of diet-induced MASLD as well [12, 13, 15, 21, 43, 44]. The absence of *Mlkl* or *Ripk3* resulted in a similar reduction of liver inflammation in aged mice, supporting the role of the Ripk3-Mlkl necroptosis pathway in liver inflammaging. In diet-induced MASLD models, the absence of MLKL consistently reduces liver inflammation [21, 24, 45], while outcomes for *Ripk3*^*−/−*^ mice vary depending on the dietary composition [13, 24, 46]. Reduced proinflammatory macrophages in *Mlkl*^*−/−*^ or *Ripk3*^*−/−*^ mice suggest that necroptosis promotes a proinflammatory macrophage phenotype, contributing to chronic liver inflammation. This aligns with studies showing that necroptosis inhibition decreases proinflammatory macrophages and inflammation in the liver and other tissues [15, 21, 47–50]. As HMGB1 drives proinflammatory macrophage polarization [51, 52], the reduced circulating HMGB1 in aged *Mlkl*^*−/−*^ or *Ripk3*^*−/−*^ mice suggests that necroptotic HMGB1 release could influence macrophage polarization in aging. Additionally, decreased systemic proinflammatory cytokines TNFα and IL-6 in aged *Mlkl*^*−/−*^ or *Ripk3*^*−/−*^ mice indicate a broader anti-inflammatory effect. Consistent with our findings, Tovey Crutchfield et al. (2023) reported reduced chronic inflammation in aged *Mlkl*^*−/−*^ mice based on inflammatory foci scoring in various tissues [53].

Aged *Mlkl*^*−/−*^ and *Ripk3*^*−/−*^ mice demonstrated reduced steatosis and liver fibrosis compared to their littermates, suggesting necroptosis-mediated inflammation contributes to age-related MASLD, as chronic inflammation is a known driver of MASH pathology [54–56]. Inhibition of the necroptosis pathway has been shown to reduce liver inflammation and pathology in MASH models [13, 15, 43, 57, 58]. Lower plasma ALT levels in knockout mice further indicate reduced liver injury, aligning with the protective effects of Mlkl or Ripk3 inhibition in aging liver. Consistent with previous reports showing that MLKL activation inhibits autophagy [24], the absence of *Mlkl* in old mice improved LC3-II/LC3-I ratio, a marker of autophagy. However, we cannot exclude the possibility that the observed changes reflect autophagosome accumulation within lysosomes rather than altered autophagic flux [59]. Furthermore, the observed decrease in markers of apoptosis and senescence with *Mlkl* and *Ripk3* deficiency aligns with previous studies demonstrating that RIPK3 induces apoptosis [27] and that necroptosis inhibition reduces senescence [15, 21]. These results indicate that both necroptosis-dependent and independent functions of Mlkl and Ripk3 influence age-related MASLD.

Our study is the first to examine the effects of *Mlkl* or *Ripk3* deficiency on lifespan and metabolic health in 22-month-old mice, revealing distinct impacts on both. We found that at 22 months of age, *Mlkl*^*−/−*^ mice exhibited improved insulin sensitivity, whereas *Ripk3*^*−/−*^ mice were glucose intolerant. Tovey Crutchfield et al. (2023) reported that *Mlkl*^*−/−*^ and *Ripk3*^*−/−*^ mice are not pre-diabetic or diabetic, however, this observation was based on blood glucose measurements in 6- and 12-month-old mice. Consistent with our findings, Rowchowdhury et al. (2016) reported glucose intolerance in 5-week-old *Ripk3*^*−/−*^ mice on a normal chow diet [46], and we and others have shown that the absence of* Mlkl* improves insulin sensitivity under high-fat diet conditions [20, 60]. The different effects of *Mlkl* and *Ripk3* deficiency on glucose and insulin tolerance in aged mice suggest their regulation of distinct metabolic pathways, independent of necroptosis. For instance, RIPK3 regulates lipid metabolism [43], while MLKL interacts with PIP2 to influence insulin signaling [60].

While absence of *Mlkl* did not impact lifespan, *Ripk3* deficiency reduced mean and median lifespan; however, body weights of old *Mlkl*^*−/−*^ or *Ripk3*^*−/−*^ mice were similar to their littermates. Li et al. (2017) reported lower body weight and youthful male reproductive organs in 15-month-old *Mlkl* and *Ripk3* knockout mice [61]. However, a study by Tovey Crutchfield et al. (2023) reported no change in body weight or reproductive organ morphology in 12-month-old knockout mice [53]. While our findings reveal that the genetic deletion of *Mlkl* or *Ripk3* protects against age-related liver inflammation, steatosis, and fibrosis, the impact on lifespan are more complex. Although we hypothesized that reducing chronic inflammation would extend lifespan, our data indicate that the absence of *Mlkl* had no effect on lifespan, while *Ripk3* deficiency was associated with reduced mean and median lifespan. These observations challenge the expectation that ablation of necroptosis-related genes would uniformly benefit overall aging outcomes. Necroptosis is a fundamental process with adaptive roles, such as tissue regeneration [62–64], tissue homeostasis [65], and immune modulation [66, 67]. Therefore, complete elimination of this pathway could disrupt these functions, counteracting localized benefits. It is noteworthy that only few studies have shown lifespan extension from fully ablating core processes; instead, benefits often arise from modulation, as seen with growth hormone signaling [68] or caloric restriction [69]. Thus, the absence of lifespan extension in *Mlkl*^*−/−*^ or *Ripk3*^*−/−*^ mice, despite reduced liver inflammation and metabolic improvements, suggests that aging involves complex systemic interactions beyond the liver. Although the reasons for the reduced lifespan in *Ripk3*^*−/−*^ mice are unclear, increased glucose intolerance with age [70] and pre-diabetic or diabetic conditions [71] are associated with reduced lifespan in humans. Thus, glucose intolerance and altered fuel utilization may negatively impact lifespan of *Ripk3*^*−/−*^ mice.

The findings reveal that inhibiting necroptosis through *Mlkl* and *Ripk3* knockouts drives adaptive changes in liver function, particularly enhancing lipid metabolism, cellular signaling, and immune regulation to maintain homeostasis. The strong enrichment of acyl-CoA and CoA hydrolase activities underscores the reliance on lipid turnover and energy homeostasis in the absence of necroptotic pathways. ACOX3 a peroxisomal enzyme upregulated in the absence of *Mlkl* or *Ripk3* is responsible for the initial step of β-oxidation of branched-chain fatty acids, and caloric restriction (CR) is reported to improve lipid metabolism in the liver of mice, and ACOX3 and ACNAT2 identified in our study are the two enzymes upregulated in response to CR [72, 73]. Proteins involved in complex and enzyme binding indicate compensatory immune and stress-response mechanisms, with reductions in proinflammatory cytokines (TNFα and IL-6) further supporting an anti-inflammatory effect from necroptosis inhibition. The correlation with drugs like pregnenolone carbonitrile that exerts antifibrotic effect [74] also suggests potential pharmacological avenues to modulate similar pathways, underscoring the therapeutic promise of selective necroptosis pathway inhibition in metabolic and inflammatory liver conditions.

In conclusion, this study underscores the importance of *Mlkl* and *Ripk3 *in driving liver inflammation and MASLD in aging via their necroptotic and non-necroptotic functions. The findings suggest that targeting *Mlkl* or *Ripk3* could provide therapeutic benefits in reducing age-related liver inflammation and pathology; however, Mlkl is a better candidate based on the adverse effects of Ripk3 deletion on glucose intolerance and lifespan. Considering that Ripk3 deletion in different cardiac cell types impacts atherosclerosis differently in *Apoe*^*−/−*^ mice [75], our future studies will investigate the cell type- and tissue-specific effects of Mlkl in aging.

## Limitations of the study

The study primarily focuses on the liver, which limits the understanding of how *Mlkl* or *Ripk3* deficiency affects other tissues and overall organismal aging. Aging is a systemic process, and effects observed in the liver may not reflect the impacts on other critical tissues. The study does not provide insights into how *Mlkl* or *Ripk3* deficiency affects systemic aging processes or interactions between different tissues. Additionally, detailed necropsy or pathological analyses at the time of death were not performed in this study. This lack of data limits our ability to identify the underlying causes of the shorter lifespan observed in *Ripk3*^*−*/−^ mice. Addressing these limitations in future research could provide a more comprehensive understanding of how MLKL or RIPK3 impact aging and lifespan.

## Supplementary Information

Below is the link to the electronic supplementary material.

Supplementary file1 (DOCX 686 KB)

## Data Availability

Proteomics data can be found in the MassIVE database via MSV000096299. Additional data supporting the study’s findings are provided within the manuscript and its supplementary materials. Correspondence and requests for information should be addressed to S. Deepa.
