# Supplementary material for: Impact of Mlkl or Ripk3 deletion on age-associated liver inflammation, metabolic health, and lifespan
Source: GeroScience. 2025 Feb 10;47(3):4465–83. doi: 10.1007/s11357-025-01553-5 (PMC12181141; doi:10.1007/s11357-025-01553-5)
Supplement: Supplementary file 1 — Supplementary file1 (DOCX 686 KB) [file 11357_2025_1553_MOESM1_ESM.docx]

**Supplementary information: GeroScience**

**Impact of *Mlkl* or *Ripk3* deletion on age-associated liver inflammation, metabolic health, and lifespan**

Sabira Mohammed^1,2^, Phoebe Ohene-Marfo^2^, Chao Jiang^1,2^, Zongkai Peng^3^, Nidheesh Thadathil^2^, Albert Tran^2^, Evan Nicklas^2^, Shylesh Bhaskaran^1,2^, Dawei Wang^2^, Ramasamy Selvarani^2^, Amit Sing^3^, Zhibo Yang^1,3^, Nagib Ahsan^3,4^, Sathyaseelan S. Deepa^1,2,5^*

1Stephenson Cancer Center, University of Oklahoma Health Sciences Center, Oklahoma City, Oklahoma, USA

2Department of Biochemistry & Physiology, University of Oklahoma Health Sciences Center, Oklahoma City, Oklahoma, USA

3 Department of Chemistry and Biochemistry, University of Oklahoma, Norman, OK, USA

4 Mass Spectrometry, Proteomics and Metabolomics Core Facility, Stephenson Life Sciences Research Center, The University of Oklahoma, Norman, OK, USA

5Oklahoma Center for Geroscience & Brain Aging, University of Oklahoma Health Sciences Center, Oklahoma City, Oklahoma, USA

Corresponding Author: Sathyaseelan S Deepa, Ph.D.

Stephenson Cancer Center

Department of Biochemistry and Physiology

Oklahoma Center for Geroscience & Brain Aging

University of Oklahoma Health Sciences Center

975 NE 10th Street, BRC-1368A

Oklahoma City, OK 73104, USA

E-mail: [Deepa-Sathyaseelan@ouhsc.edu](mailto:Deepa-Sathyaseelan@ouhsc.edu)


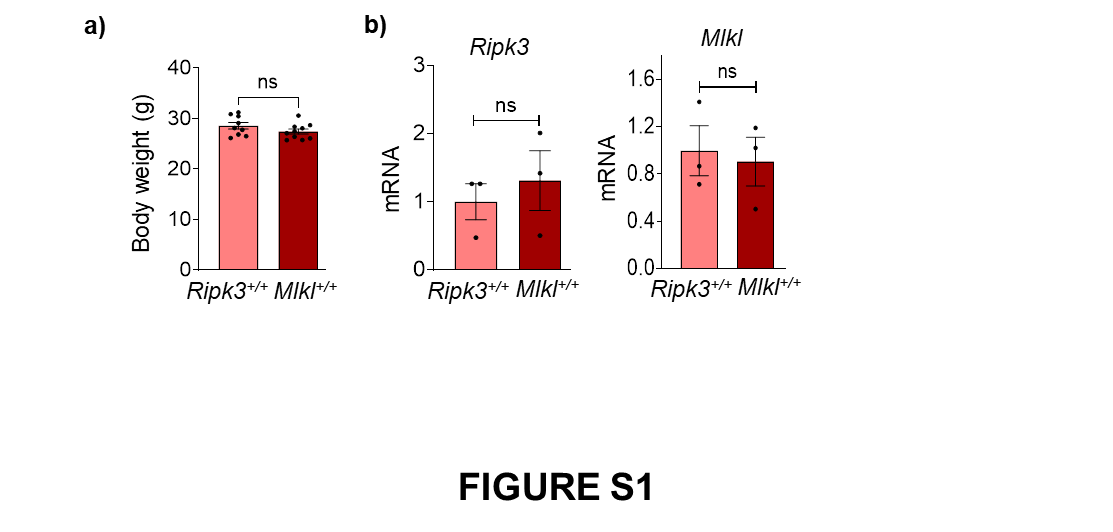


**Fig. S1 (**a) Body weight of *Ripk3^+/+^* and *Mlkl^+/+^* mice used for the study measured at 3 months of age (b) Transcript levels of *Mlkl* and *Ripk3* in the livers of *Ripk3^+/+^* and *Mlkl^+/+^* mice measured at 24 months of age. Error bars are represented as mean ± SEM. Unpaired t-test, ns: p>0.05. (F value, p value): (a) 1.55, 0.522; (b, *Mlkl*) 1.05, 0.97; (b, *Ripk3*) 2.76, 0.53

**
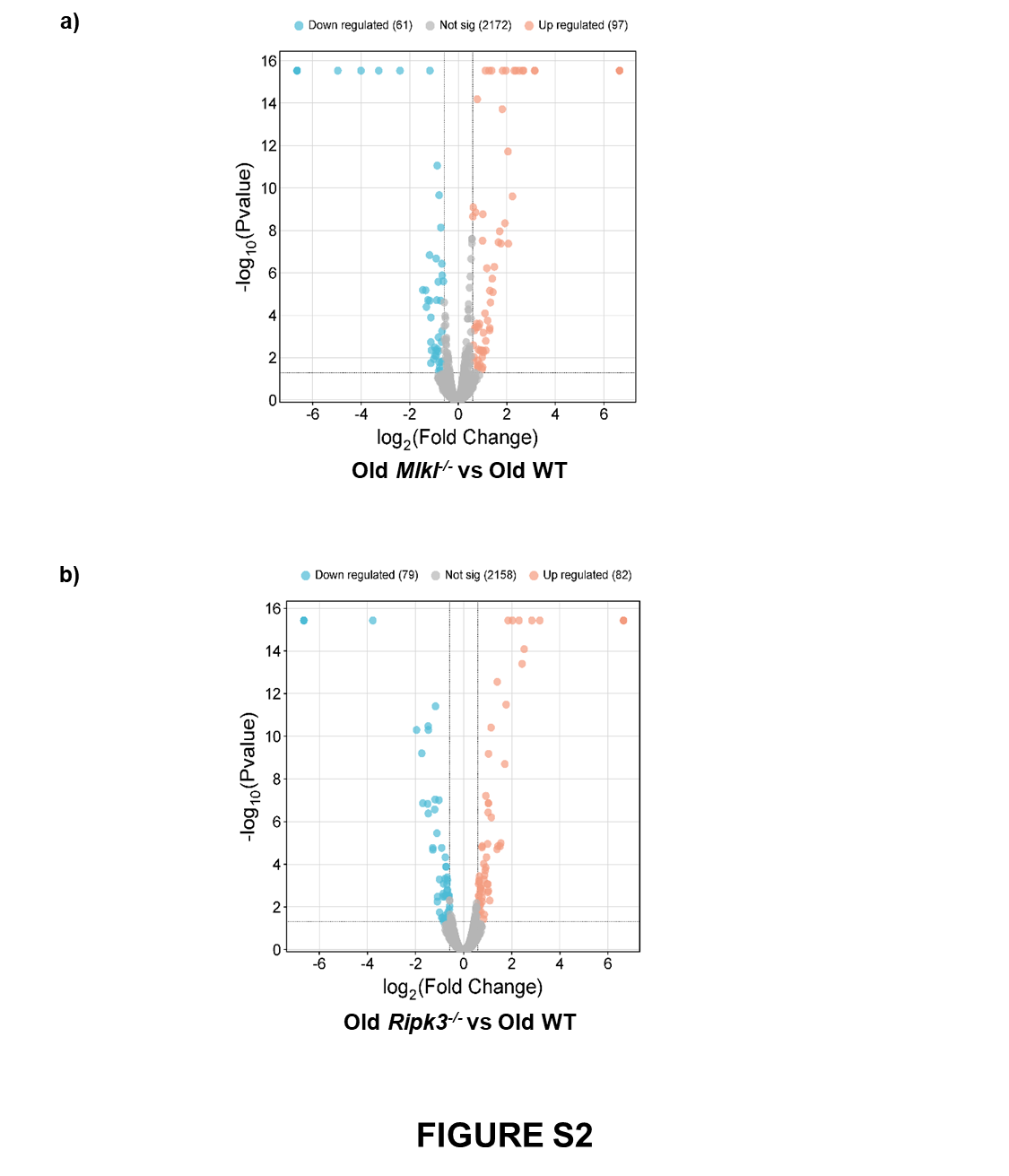
**

**Fig. S2** Volcano plot analysis of significantly modulated proteins. (a) old *Mlkl^-/-^* mice and (b) old *Ripk3^-/-^* mice when compared to old WT. Significant proteins represented with a fold change of at least 1.5 with an adjusted p-value < 0.05. N=5 per group


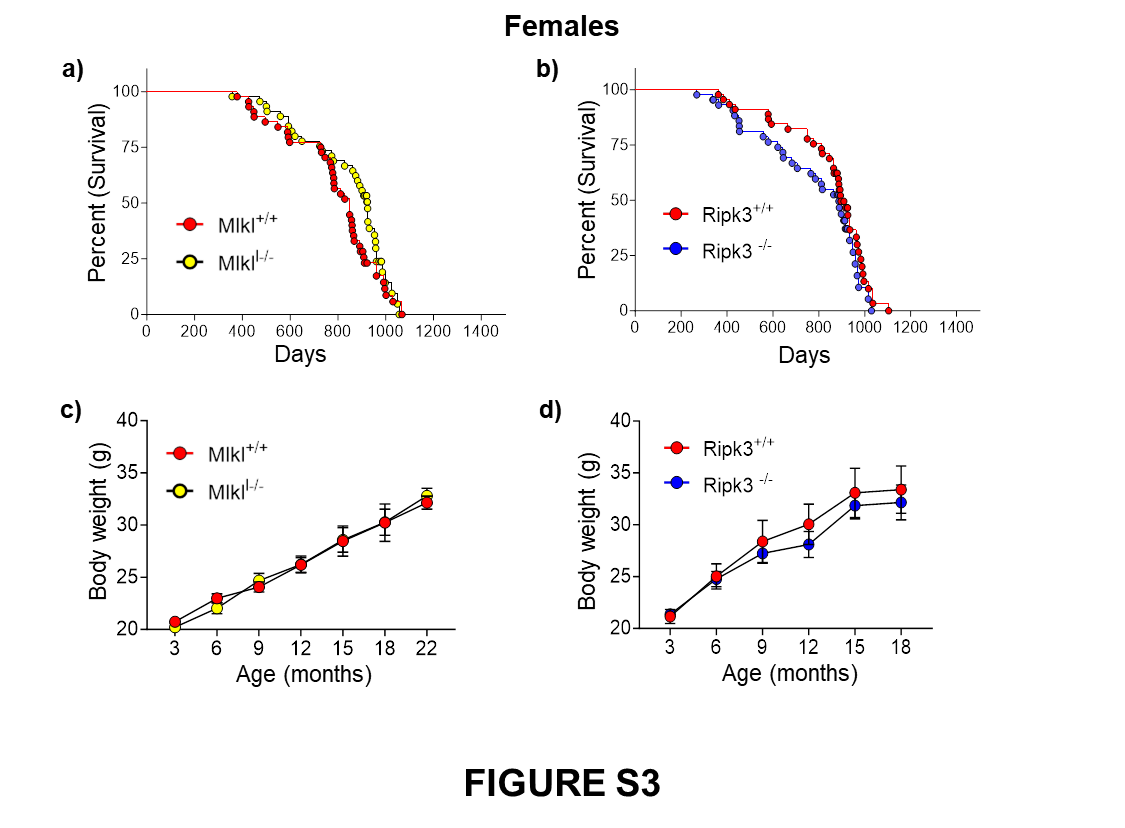


**Fig. S3** Effect of absence of *Ripk3 or Mlkl* on survival and body weight of female mice. Kaplan-Meier survival curve for (a) *Mlkl^+/+^, Mlkl^-/-^* (on the left) or (b) *Ripk3^+/+^, Ripk3^-/-^* (on the right). Average body weight changes of (c) *Mlkl^+/+^* , or *Mlkl^-/-^* and (d) *Ripk3^+/+^ , Ripk3^-/-^* mice. a, b: n= 44 *Mlkl^+/+^*, n=45 *Mlkl^-/-^ ,* n=45 *Ripk3^+/+^*, n=44 *Ripk3^-/-^*. c, d: n=10/ group. Data are expressed as mean ± SEM and analyzed using a two-way ANOVA. ANOVA summary (F value, p value): (c) row factor (time points) 42.53, <0.0001 column factor (genotype) 0.00038, 0.995; (d) row factor (time points) 18.61, <0.0001 column factor (genotype) 1.21, 0.273

**
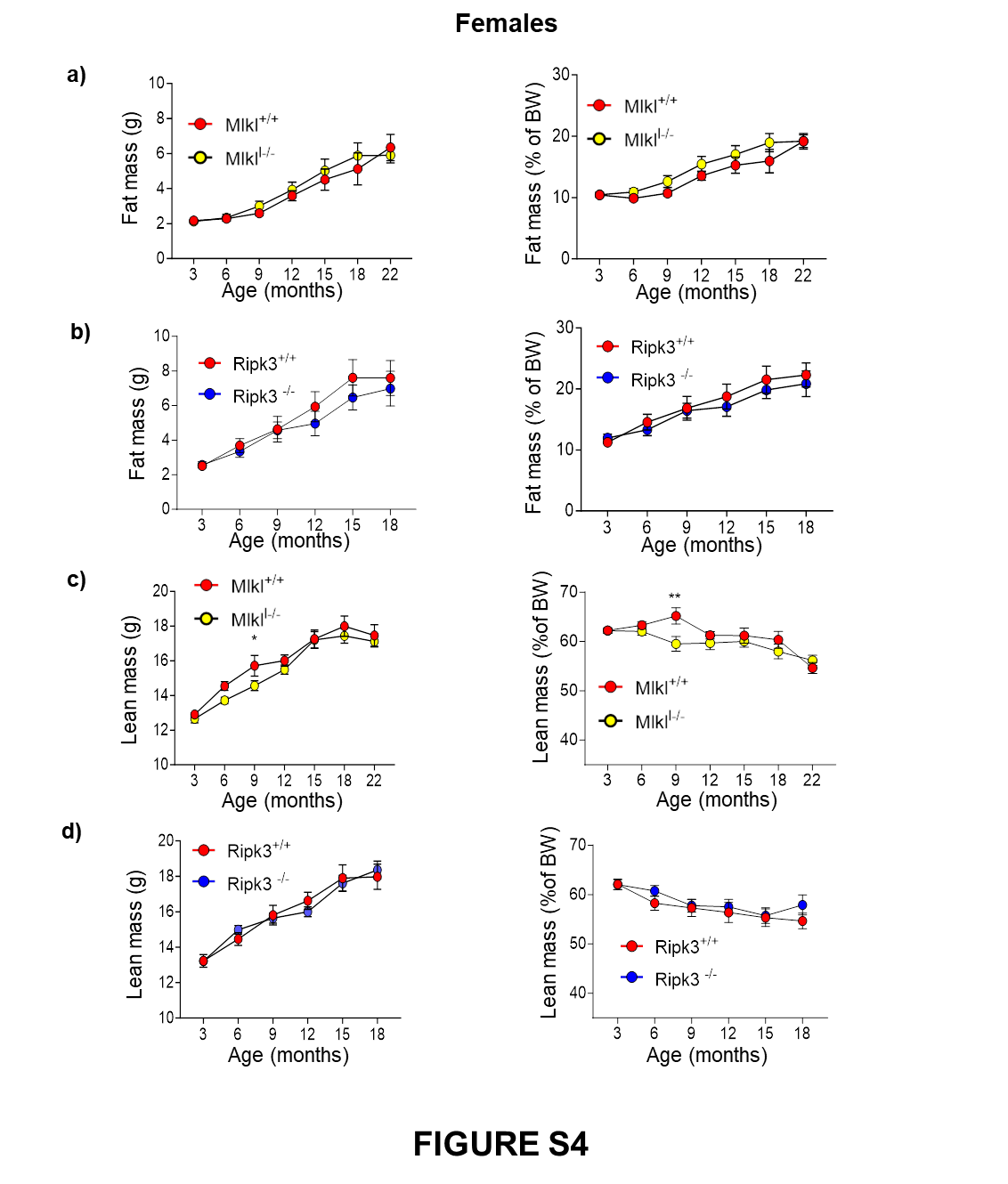
**

**Fig. S4** Effect of absence of *Ripk3 or Mlkl* on body composition of female mice. Gross fat mass (in grams) (on the left) and percentage of fat mass normalized to total body weight (on the right) of (a) *Mlkl^+/+^*, *Mlkl^-/-^* mice and (b) *Ripk3^+/+^, Ripk3^-/-^* mice. Gross lean mass (in grams) (on the left) and percentage of lean mass normalized to total body weight (on the right) of (c) *Mlkl^+/+^*, *Mlkl^-/-^* mice and (d) *Ripk3^+/+^, Ripk3^-/-^* mice. n=10/group. Data are expressed as mean ± SEM and analyzed using a two-way ANOVA. * p< 0.05, ** p< 0.005, *** p<0.0005. ANOVA summary (F value, p value): (a, left) row factor (time points) 21.78, <0.0001 column factor (genotype) 0.78, 0.38; (a, right) row factor (time points) 20.85, <0.0001 column factor (genotype) 5.56, 0.02; (b, left) row factor (time points) 15.13, <0.0001 column factor (genotype) 1.65, 0.20; (b, right) row factor (time points) 12.16, <0.0001 column factor (genotype) 1.11, 0.295; (c, left) row factor (time points) 42.2, <0.0001 column factor (genotype) 6.09, 0.015; (c, right) row factor (time points) 8.5, <0.0001 column factor (genotype) 5.27, 0.023; (d, left) row factor (time points) 34.67, <0.0001 column factor (genotype) 0.015, 0.90; (d, right) row factor (time points) 4.92, 0.005 column factor (genotype) 2.06, 0.154


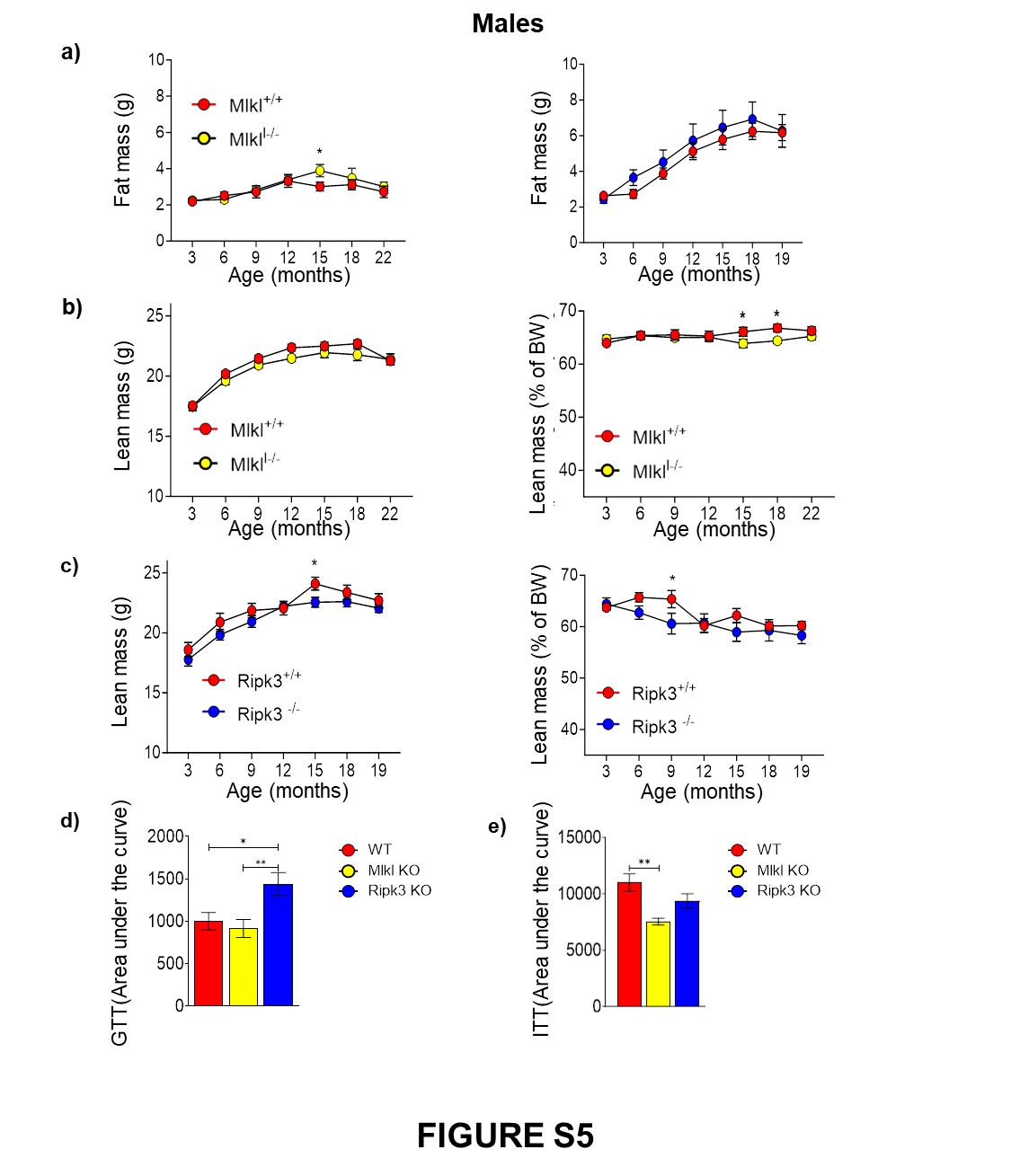


**Fig. S5** Effect of absence of *Ripk3 or Mlkl* on body composition of male mice. (a) Gross fat mass (in grams) *Mlkl^+/+^*, *Mlkl^-/-^* mice (on the left) and *Ripk3^+/+^, Ripk3^-/-^* mice (on the right). (b) Gross

lean mass (in grams) (on the left) and percentage of lean mass normalized to total body weight (on the right) of *Mlkl^+/+^*, *Mlkl^-/-^* mice (c) Gross lean mass (in grams) (on the left) and percentage

of lean mass normalized to total body weight (on the right) of *Ripk3^+/+^, Ripk3^-/-^* mice. Area under the curve for (d) GTT represented in Figure 6g and (e) ITT represented in Figure 6h. ANOVA summary (F value, p value): (a, left) row factor (time points) 6.04, <0.0001 column factor (genotype) 2.17, 0.143; (a, right) row factor (time points) 13.72, <0.0001 column factor (genotype) 2.2, 0.140; (b, left) row factor (time points) 55.22, <0.0001 column factor (genotype) 7.752, 0.006; (b, right) row factor (time points) 0.73, 0.63 column factor (genotype) 3.98, 0.048; (c, left) row factor (time points) 22.66, <0.0001 column factor (genotype) 7.750, 0.0063; (c, right) row factor (time points) 3.87, 0.0015 column factor (genotype) 4.87, 0.029; (d) 5.38, 0.012; (e) 6.87, 0.006

**TABLE S1** List of proteins identified by label-free quantitative proteomic analysis of liver tissues from young WT, old WT, old *Mlkl^-/^*^-^, and old *Ripk3^-/-^* mice.

| **Proteins commonly upregulated in old *Mlkl^-/-^* and old *Ripk3^-/-^*** | **Proteins commonly downregulated in old *Mlkl^-/-^* and old *Ripk3^-/-^*** |
| --- | --- |
| UBXN1  TSNAX  STX8  SF3B4  RPL37  PTRHD1  PCNA  MTMR3  LRG1  IQGAP2  IPO9  FCER1G  FAM114A1  ERLIN2  CSRP2  CD74  CAMK2A  AFDN  ACNAT2  PDGFC  IGSF9B  MUP17  SLCO2B1  ACOT3  ABHD12  NUCKS1  ABLIM1  MRPL50  RNF185  SELENBP2  TOMM5  DIAPH1 | PKLR  H1-5  UGT1A9  CD81  NAPA  S100A11  IFGGD1  UBE2L6  NT5C  U2AF2  ARL1  JPT2  ZC3H15  TBC1D5  SHFL  LYPLA2  ITPRID2  ERBIN  DSC2  DHRS3  DDX21 |

**TABLE S2** Sequences of quantitative PCR primers used in the study.

| **Gene** | **Forward sequence** | **Reverse sequence** |
| --- | --- | --- |
| Acta2 | 5’-CTGACAGAGGCACCACTGAA-3’ | 5’-CATCTCCAGAGTCCAGCACA-3’ |
| Arg1 | 5’-CTCCAAGCCAAAGTCCTTAGAG-3’ | 5’-AGGAGCTGTCATTAGGGACATC-3’ |
| β- actin | 5′-ATGGATGACGATATCGCTG-3′ | 5′-GTTGGTAACAATGCCATGTTC-3′ |
| β-microglobulin | 5′-CACTGACCGGCCTGTATGC-3′ | 5′-GGGTGGCGTGAGTATACTTGAAT-3′ |
| Cdkn2a (p16^Ink4a^) | 5’-CCCAACGCCCCGAACT-3’ | 5’-GCAGAAGAGCTGCTACGTGAA-3’ |
| Cdkn1a (p21^Cip1^) | 5’-GTCAGGCTGGTCTGCCTCCG-3’ | 5’-CGGTCCCGTGGACAGTGAGCAG-3’ |
| Col1α1 | 5’-GCTCCTCTTAGGGGCCACT-3’ | 5’-CCACGTCTCACCATTGGGG-3’ |
| CD11c | 5’-CTGGATAGCCTTTCTTCTGCTG-3’ | 5’-GCACACTGTGTCCGAACTC-3’ |
| CD68 | 5’-CCACAGGCAGCACAGTGGAC-3’ | 5’-TCCACAGCAGAAGCTTTGGCCC-3’ |
| CD86 | 5’-ACGATGGACCCCAGATGCACCA-3’ | 5’-GCGTCTCCACGGAAACAGCA-3’ |
| CXCL2 | 5’-CCTGGTTCAGAAAATCATCCA-3’ | 5’-CTTCCGTTGAGGGACAGC-3’ |
| Fizz1 | 5’-CCAATCCAGCTAACTATCCCTCC-3’ | 5’-CCAGTCAACGAGTAAGCACAG-3’ |
| HPRT | 5’-CTGGTGAAAAGGACCTCTCG-3’ | 5’-TGAAGTACTCATTATAGTCAAGGGCA-3’ |
| IL1β | 5’-AGGTCAAAGGTTTGGAAGCA-3’ | 5’-TGAAGCAGCTATGGCAACTG-3’ |
| IL-6 | 5’-TGGTACTCCAGAAGACCAGAGG-3’ | 5’-AACGATGATGCACTTGCAGA-3’ |
| MCP1 | 5’-TTAAAAACCTGGATCGGAACCAA-3’ | 5’-GCATTAGCTTCAGATTTACGGGT-3’ |
| MLKL | 5′-CTGAGGGAACTGCTGGATAGAG-3′ | 5′-CGAGGAAACTGGAGCTGCTGAT-3′ |
| MMP3 | 5’-GTTGGAGAACATGGAGACTTTGT-3’ | 5’-CAAGTTCATGAGCAGCAACCA-3’ |
| MMP12 | 5’-TGCACTCTGCTGAAAGGAGTCT-3’ | 5’-GTCATTGGAATTCTGTCCTTTCCA-3’ |
| P19 | 5'-GGGTCGCAGGTTCTTGGTC-3' | 5'-AATCTGCACCGTAGTTGAGCA-3' |
| RIPK3 | 5′-GAAGACACGGCACTCCTTGGTA-3′ | 5′-CTTGAGGCAGTAGTTCTTGGTGG-3′ |
| TGFβ | 5’-ACCATGCCAACTTCTGTCTGGGAC-3’ | 5’-ACAACTGCTCCACCTTGGGCTTG-3’ |
| TNFα | 5’-CACAGAAAGCATGATCCGCGACGT-3’ | 5’- CGGCAGAGAGGAGGTTGACTTTCT-3’ |

**Impact of *Mlkl* or *Ripk3* deletion on age-associated liver inflammation, metabolic health, and lifespan**

Sabira Mohammed^1,2^, Phoebe Ohene-Marfo^2^, Chao Jiang^1,2^, Zongkai Peng^3^, Nidheesh Thadathil^2^, Albert Tran^2^, Evan Nicklas^2^, Shylesh Bhaskaran^1,2^, Dawei Wang^2^, Ramasamy Selvarani^2^, Amit Singh^3^, Zhibo Yang^1,3^, Nagib Ahsan^3,4^, Sathyaseelan S. Deepa^1,2,5^*

^1^Stephenson Cancer Center, University of Oklahoma Health Sciences Center, Oklahoma City, Oklahoma, USA

^2^Department of Biochemistry & Physiology, University of Oklahoma Health Sciences Center, Oklahoma City, Oklahoma, USA

^3^ Department of Chemistry and Biochemistry, University of Oklahoma, Norman, OK, USA

^4^ Mass Spectrometry, Proteomics and Metabolomics Core Facility, Stephenson Life Sciences Research Center, The University of Oklahoma, Norman, OK, USA

^5^Oklahoma Center for Geroscience & Brain Aging, University of Oklahoma Health Sciences Center, Oklahoma City, Oklahoma, USA

*Corresponding Author: Sathyaseelan S Deepa, Ph.D.

Stephenson Cancer Center

Department of Biochemistry and Physiology

Oklahoma Center for Geroscience & Brain Aging

University of Oklahoma Health Sciences Center
975 NE 10th Street, BRC-1368A
Oklahoma City, OK 73104, USA

E-mail: Deepa-Sathyaseelan@ouhsc.edu

Phone: 405-271-8001 (Extn:48393)

Fax: 405-271-2298

Running title: Mlkl/Ripk3 deletion in aging and liver health

**Abstract**

Chronic, low-grade inflammation is a hallmark of aging and various age-related diseases, including metabolic dysfunction-associated steatotic liver disease (MASLD). The prevalence of metabolic dysfunction-associated steatohepatitis (MASH), an advanced form of MASLD, increases with age and contributes to morbidity and mortality among the elderly. This study investigates the role of necroptosis, a programmed cell death pathway that promotes inflammation, in liver inflammaging and age-associated MASLD by utilizing genetic ablation models of two key necroptosis proteins, Mlkl or Ripk3. Absence of Mlkl or Ripk3 significantly reduced liver inflammation, steatosis, and fibrosis in aged male mice, supporting a role of necroptosis in age-associated MASLD. Additionally, Mlkl or Ripk3 deletion impacted other non-necroptotic cellular processes that drive inflammation and MASLD, such as cellular senescence, apoptosis, and autophagy in aged liver. Levels of plasma TNFα and IL6, key proinflammatory cytokines associated with inflammaging, are reduced in *Mlkl^-/-^* or *Ripk3^-/-^* aged mice, supporting a systemic effect of necroptosis inhibition on inflammation. Proteomic analysis of liver tissues emphasizes the critical role of lipid and immune regulatory processes in maintaining liver homeostasis when Mlkl or Ripk3 is absent in aging liver. While Mlkl deletion did not affect the lifespan of mice, Ripk3 deletion shortened it. Additionally, Mlkl deficiency improved insulin sensitivity, whereas Ripk3 deficiency exacerbated glucose intolerance in aged mice. Thus, selective inhibition of Mlkl, not Ripk3, represents a potential therapeutic avenue for mitigating age-related liver disease and enhancing metabolic outcomes in the elderly.

**Key words**: Inflammaging, liver, Ripk3, Mlkl, lifespan, MASLD

**Introduction**

Chronic, low-grade, sterile inflammation, termed "inflammaging," is recognized as one of the "seven pillars of aging"[1, 2]. In humans, it is marked by increased circulating pro-inflammatory cytokines like IL-6, TNF-α, and IL-1β, which are linked to higher disease and mortality rates [3]. Age-related diseases such as cardiovascular disease, cancer, diabetes, and neurodegenerative disorders are associated with a persistent inflammatory state [4, 5]. Thus, chronic inflammation is a key factor in aging and age-related diseases [1].

Chronic inflammation in the aging liver, or liver inflammaging, is linked to metabolic dysfunction-associated steatotic liver disease (MASLD), a spectrum of liver disease conditions from simple steatosis to metabolic dysfunction-associated steatohepatitis (MASH), characterized by hepatocyte ballooning, inflammation, and fibrosis. MASLD/MASH prevalence nearly doubles in individuals aged 45-64 compared to those aged 20-44 and is associated with higher mortality in people aged 60-74 [6, 7]. MASH is a major risk factor for hepatocellular carcinoma (HCC) [8] and cardiovascular diseases [9] and is now the leading indication for liver transplantation in the elderly in the U.S., with the proportion of elderly patients requiring transplants rising from 9% (2002-2005) to 23% (2018-2020) [10]. Despite many clinical trials, Resmetirom remains the only FDA-approved drug for MASH (March 2024) [11], underscoring the need to better understand MASLD/MASH pathogenesis in aging.

Chronic inflammation is a key mechanism in MASLD development, making inflammaging a target for preventing age-related MASLD. Studies show that necroptosis, an inflammatory cell death pathway, contributes to liver inflammation and MASH in diet-induced MASLD/MASH mouse models [12, 13]. Necroptosis is triggered by stimuli (e.g., oxidative stress, TNFα), which sequentially activate receptor interacting serine/threonine kinase 1 (Ripk1), Ripk3, and mixed lineage kinase domain-like (Mlkl) through phosphorylation, which then permeabilizes the membrane and releases DAMPs. DAMPs activate immune cells, increasing cytokine production and creating a feedback loop of inflammation and cell death [14]. Our group has shown that necroptosis markers increase with age in mouse livers, correlating with inflammation and MASH [15], and are elevated in the superoxide dismutase 1 knockout (*Sod1^-/-^*) mice, an accelerated aging model [16]. Treating aged WT or adult *Sod1^-/-^* mice with necrostatin-1s, a RIPK1 inhibitor, reduces liver inflammation and MASH pathology, underscoring the necroptosis pathway’s role in age-related liver inflammation and MASH.

An increase in liver necroptosis markers correlates not only with liver inflammaging but also with lifespan in mice. While natural aging increases markers of necroptosis and inflammation in the livers of mice, these markers are significantly downregulated in the livers of aged Ames Dwarf mice, a mouse model of extended lifespan [17]. Conversely, markers of necroptosis and inflammation are increased in the livers of young *Sod1^-/-^* mice that has reduced lifespan [16]. Therefore, to gain a better understanding of the role of necroptosis in liver inflammaging, age-associated MASLD, and lifespan, we genetically inhibited two key proteins in the necroptosis pathway, *Mlkl* or *Ripk3*, in mice. Our results show that absence of *Mlkl* or *Ripk3* reduced liver inflammaging and age-associated MASLD pathology, however, absence of these proteins had differential effects on lifespan and metabolic health. Additionally, the absence of *Mlkl* or *Ripk3* affected various cellular mechanisms involved in inflammation, including cellular senescence, autophagy, and apoptosis. These data indicate that Mlkl and Ripk3 have non-necroptotic roles, in addition to necroptosis, in regulating key pathways linked to inflammaging in the livers of aged mice.

**Methods**

**Animals**

All procedures were conducted according to the protocol approved by the Institutional Animal Care and Use Committee at the University of Oklahoma Health Sciences Center (OUHSC). *Ripk3^-/-^* mice were obtained from Genentech (South San Francisco, CA, USA) [18] and *Mlkl^-/-^* mice were provided by Dr. James Murphy (Walter and Eliza Hall Institute of Medical Research, Australia) [19]. We used *Ripk3^+/-^* or *Mlkl^+/-^* mice solely for breeding to generate *Ripk3^+/+^*, *Ripk3^-/-^*, *Mlkl^+/+^*, and *Mlkl^-/-^* mice, and all mice were in C56BL/6J background. Heterozygous mice produced during breeding were not included in the experiments. Mice were rehoused to form groups of wild-type and knockout mice from different litters. The mice were group housed in ventilated cages at 20°C ± 2°C and were fed with normal chow diet (5053 Pico Lab, Purina Mills, Richmond, IN) on a 12-hour dark/light cycle. For the lifespan study using male mice, we used 40 *Ripk3^+/+^*, 42 *Ripk3^-/-^*, 42 *Mlkl^+/+^*, 45 *Mlkl^-/-^* mice. Similarly, for the lifespan study using female mice, 45 *Ripk3^+/+^*, 44 *Ripk3^-/-^*, 44 *Mlkl^+/+^*, 45 *Mlkl^-/-^* mice were used. For survival analysis, the mice were allowed to live out their natural lifespan and the time of their spontaneous death and the ages were recorded. The mean, median, percentiles (10%, 25%, 75%, 90%), maximum lifespan for each group was calculated. For analyzing the glucose tolerance, insulin tolerance and body composition, a separate cohort comprising of n=10 mice/group was maintained. Body weight, lean mass and fat mass were assessed every month by using the Quantitative Magnetic Resonance method (Bruker minispec LF90, MA, USA). For all other studies, we used liver tissue from mice that were generated and housed at the Oklahoma City Veterans Affairs Health Care System Animal Facility, and all procedures were approved by the Institutional Animal Care and Use committee at the Oklahoma City Veterans Affairs Health Care System Animal Facility. For studies involving aged liver, we used young wild type (WT, 7 months) and old (24 -25 months; WT, *Mlkl^-/-^, and Ripk3^-/-^*) male mice. The WT mice represent a mix of *Ripk3^+/+^* and *Mlkl^+/+^*mice.

**Western Blotting**

Western blot analysis was performed as described previously[20]. The following primary antibodies were used: MLKL (Millipore Sigma, Burlington, MA), RIPK3 (Novus Biologicals, Centennial, CO), LC3I/II (Cell Signaling Technology, Danvers, MA), β-actin (Sigma-Aldrich, St. Louis, MO). HRP-linked secondary antibodies were from Cell Signaling Technology. Images were taken with the Chemidoc imager (Bio-Rad) and quantified with ImageJ software (U.S. National Institutes of Health).

**Immunohistochemistry (IHC) staining**

IHC staining was performed using paraffin embedded liver sections for P-MLKL (Abcam, Cambridge, UK), F4/80 (Proteintech, Rosemont, IL) and Cleaved Caspase 3 (Cell Signaling Technology) using a standardized protocol [21]. Images were taken using an ECHO REVOLVE R4 microscope for three random non-overlapping fields per sample. For P-MLKL, staining intensity was quantified using ImageJ software by using the color deconvolution plug-in and percentage area of DAB (3, 3'-diaminobenzidine) staining was obtained for quantification. For F4/80 and Cleaved Caspase 3 staining, the number of positively stained cells per field was quantified using the Cell Count feature in the Echo Revolve R4 microscope. Three random fields per sample were acquired.

**Quantitative real-time PCR (RT-PCR)**

RNA was isolated from 20 mg frozen liver tissues, and the real-time-PCR was performed as described previously [20]. The calculations were performed by a comparative method (2^−ΔΔCt)^ using β-microglobulin, β-actin, or hypoxanthine phosphoribosyltransferase 1 (HPRT) as housekeeping genes. The data are represented as fold change after normalization to the young WT mice group. The primers used are listed in Table S2.

**Picrosirius red (PSR) staining**

PSR staining was done using a standard protocol [21]. The images were taken using an ECHO REVOLVE R4 microscope for 3 random non-overlapping fields per sample. The percentage area of PSR staining was quantified using the thresholding option in ImageJ software.

**Histological Analysis**

Formalin-fixed liver tissue was embedded in paraffin and sectioned. The sections were then stained with Hematoxylin & Eosin (H&E) using a standard protocol [21]. Images were taken using an ECHO REVOLVE R4 microscope for 3 random non-overlapping fields per sample. The hepatic steatosis was quantified using the Cell Count option in Echo Revolve R4 microscope and is represented graphically.

**Quantification of liver triglyceride**

Liver triglyceride levels were quantified using a triglyceride colorimetric assay kit from Cayman Chemical Company (Ann Arbor, MI, USA) as described [20], following manufacturer’s instructions.

**Glucose Tolerance (GTT) and Insulin Tolerance Tests (ITT)**

GTT and ITT were performed as described before [20]. Briefly, mice were fasted for 6 hours for GTT and received intraperitoneal injection of glucose (2g/kg, Sigma Aldrich) or 5 hours for ITT and received insulin (0.75 units/kg, Novo Nordisk Inc., Bagsvaerd, Denmark). Blood glucose concentration was measured before glucose or insulin injection and then 15, 30, 60, and 120 minutes after administration using TRUE METRIX glucose strips and glucometer (Trividia Health Inc., Plainsboro Township, NJ, USA).

**Plasma analyses for high-mobility group box-1(HMGB1), Alanine Transaminase (ALT) and pro-inflammatory cytokines**

The levels of ALT and HMGB1 in plasma were measured using ALT colorimetric activity assay kit from Cayman Chemical Company and mouse HMGB1 ELISA Kit (Elabscience, Houston, TX) as per manufacturer’s instructions. The pro-inflammatory cytokines in plasma were determined using Meso Scale Discovery V-PLEX Custom Mouse Biomarkers Proinflammatory Panel1 (K152A0H-1, MSD, Rockville, MD).

**Proteomic analysis of liver samples**

A total of 100 µg of liver proteins (n=5/group) were subjected for in-solution trypsin/LysC (Cat# V5071, Promega, WI, USA) digestion. Trypsin digestion was performed according to the manufacturer protocol. Following digestion, the peptides were desalted using C18 Sep-Pak Plus cartridges (Waters, MA, USA). The dried tryptic peptides were reconstituted with 100 µL of 0.1% formic acid to a final concentration of 1 µg/µL. The resuspended tryptic peptides (2 μL) were loaded onto a C18 trap column (150 μm × 3 cm, 3 μm resin, Acclaim™ PepMap™ 100 C18 HPLC Column, Thermo Scientific™, USA) using mobile phase A (0.1% formic acid in LC-MS grade water) at a flow rate of 3 μl/min for 10 min, and separate peptides on an EASY-Spray™ HPLC analytical column (3 μm x 75 μm × 15 cm, Catalog # ES900 Thermo Scientific™, USA) at 350 nL/min. The total LC-MS/MS run time is 60 min, including column wash and re-equilibration. The LC-MS/MS analysis was conducted using a Dionex UltiMate® 3000 UHPLC system (Thermo Fisher Scientific, CA, USA) coupled to a Q Exactive HF-X mass spectrometer (Thermo Fisher Scientific, Waltham, MA) as described previously [22].

The RAW MS files were searched against the UniProt reviewed mouse (Taxon ID: 10090) protein database using the Sequest algorithm within Proteome Discoverer v 2.4 (Thermo Fisher Scientific, San Jose, CA). Parameters used for the Sequest database search are listed as follows: trypsin enzyme cleavage specificity, 2 possible missed cleavages, 10 ppm mass tolerance for precursor ions, and 0.02 Da mass tolerance for fragment ions. Search parameters permit dynamic modification of methionine oxidation (+15.9949 Da) and static modification of carbamidomethylation (+57.0215 Da) on cysteine. Peptide assignments from the database search are filtered down to a 1% FDR (false discovery rate). Label-free quantitation across the samples employs the Minora algorithm and the adjoining bioinformatics tools available in Proteome Discoverer. A 1.5-fold increase or decrease in abundance with a p-value <0.05 is considered statistically significant. Proteomics data can be found in the MassIVE database via MSV000096299.

**Bioinformatics**

Heatmaps, and volcano plots, were generated by SRplot (<https://www.bioinformatics.com.cn/en>), a free online platform for data analysis and visualization. Venn diagram was generated using an open-source platform (https://pnnl-comp-mass-spec.github.io/Venn-Diagram-Plotter/). All pathway analyses were performed using ShinyGO 0.80 bioinformatics platform[23]. The remaining parts were generated by Microsoft Office PowerPoints and Excel 365.

**Statistical analysis**

All data are represented as mean±SEM. Ordinary one-way ANOVA with uncorrected Fisher’s LSD test was used to analyze data with GraphPad Prism. F-values and p-values from the ANOVA summary are included in the figure legends to provide a complete representation of the statistical analysis. For survival curve analysis, the mean, median and percentile values were obtained by performing descriptive statistics analysis for the data simple survival analysis (Kaplan-Meier) using GraphPad Prism. Mann Whitney test was performed on the data for statistical significance. The significance between the survival curves was analyzed by performing Mantel-Cox test followed by Gehan-Breslow-Wilcoxon test. P< 0.05 is considered statistically significant.

**Results**

**Absence of *Mlkl* or *Ripk3* reduced liver inflammation in aged mice**

To evaluate the impact of *Mlkl* or *Ripk3* deficiency on age-associated MASLD, we used *Mlkl^-/-^* or *Ripk3^-/-^* male mice. A combination of *Mlkl^+/+^* or *Ripk3^+/+^* aged mice were used as old wild-type (WT) controls, as our data show that the body weight and expression of *Mlkl* or *Ripk3* are similar in both *Mlkl^+/+^* and *Ripk3^+/+^* mice (Fig S1a, b). The liver weight remained unchanged between the study groups (Figure 1a). However, the liver weight, when normalized to body weight, was significantly higher in old WT mice compared to young mice, whereas the absence of *Mlkl* or Ripk3 significantly reduced liver weight in aged mice (Figure 1a). Protein expression and transcript levels of Mlkl (3.4 -fold and 1.3-fold) and Ripk3 (4.6-fold and 2.5-fold) were significantly elevated in the livers of old WT mice, compared to young WT mice (Figures 1b-c). Absence of Mlkl reduced Ripk3 expression, and vice versa, at both transcript and protein levels. (Figures 1b-c). Measurement of phosphorylated Mlkl (P-Mlkl), a marker of necroptosis, by immunostaining of liver tissues showed a 20-fold increase in P-MLKL staining in the livers of old WT mice compared to young WT mice, while both *Mlkl^-/-^* and *Ripk3^-/-^* old mice showed significant reduction in P-Mlkl staining (Figure 1d). The circulating level of HMGB1, a pro-inflammatory DAMP released during necroptosis, was significantly increased in the plasma of old WT mice and was reduced in both aged knockout mice (Figure 1e).

Immunostaining for the macrophage marker, F4/80, showed that aging resulted in a significant increase in the number of F4/80 positive cells in the liver (1.9-fold) (Figure 2a). Additionally, markers of proinflammatory macrophages CD11c, CD86 and CD68 (2.6-fold, 1.9-fold, 1.7-fold) were significantly upregulated, and markers of anti-inflammatory macrophages Arg1 (0.6-fold) and Fizz1 (0.4-fold) were significantly downregulated in old WT mice relative to young mice (Figure 2b). Absence of Mlkl or Ripk3 significantly reduced the number of F4/80 positive cells and proinflammatory macrophage markers in aged mice, however anti-inflammatory macrophage markers were unaffected by the absence of either Mlkl or Ripk3. Consistent with the increased levels of proinflammatory macrophage markers, the transcript levels of pro-inflammatory cytokines associated with inflammaging (TNFα, IL6, and IL1β) and the chemokine MCP1 were significantly upregulated in the livers of old WT mice compared to young mice (TNFα: 4.3-fold; IL6: 3.7-fold; IL1β: 4.4-fold; MCP1: 11.8-fold), and absence of *Mlkl* or *Ripk3* significantly reduced their levels in aged mice (Figure 2c). The levels of circulating TNFα (1.9-fold) and IL6 (3.3-fold) were significantly elevated in old WT mice relative to young mice, while *Mlkl^-/-^* or *Ripk3^-/-^* old mice showed a significant reduction in their levels compared to aged WT mice (Figure 2d). Thus, our data show that absence of *Mlkl* or *Ripk3* mitigates age-related proinflammatory macrophage accumulation and hepatic inflammation.

**Aged *Mlkl^-/-^* or *Ripk3^-/-^* mice exhibited reduced steatosis and liver fibrosis**

H&E staining of liver tissues showed that aging-induced microvesicular steatosis was significantly reduced in both *Mlkl^-/-^* and *Ripk3^-/-^* old mice (Figure 3a). In line with this, hepatic triglyceride content was 1.4-fold higher in old WT mice compared to young mice, while the absence of *Mlkl* or *Ripk3* significantly reduced triglyceride levels in aged mice (Figure 3b). Assessment of liver fibrosis using PSR staining that detects collagen fibers in a tissue revealed a significant increase in PSR staining in old WT mice (7.3-fold) compared to young mice, whereas *Mlkl^-/-^* or *Ripk3^-/-^* aged mice showed a significant reduction in PSR staining (Figure 3c). This observation was consistent with transcript levels of fibrosis markers, which were significantly elevated in old WT mice (Acta2: 2.2-fold; Col1α1: 1.7-fold) compared to young mice, while absence of *Mlkl* or *Ripk3* significantly reduced their levels in aged mice (Figure 3d). Additionally, plasma ALT level, an indicator of liver injury, increased by nearly 3.3-fold in old WT mice but was significantly reduced in the absence of *Mlkl* or *Ripk3* (Figure 3e). Thus, the absence of *Mlkl* or *Ripk3* protects mice against age-related MASLD/MASH pathology by reducing liver steatosis, fibrosis, and liver injury.

**Absence of Mlkl or Ripk3 reduced cellular senescence markers in aged liver**

Our previous study has shown that pharmacological inhibition of necroptosis with necrostatin-1s reduced markers of cellular senescence in the livers of old WT mice [15]. Based on these findings, we investigated the effect of genetic inhibition of necroptosis pathway proteins on cellular senescence in aged liver. Markers of cellular senescence (p16: 9.5-fold, p21: 8.2-fold, p19: 2.5-fold) and senescence associated secreted factors, SASP (TGFβ: 2-fold, MMP12: 40-fold, CXCL2: 10-fold) were significantly upregulated in the livers of old WT mice compared to young mice, except for MMP3 (Figure 4a). The absence of *Mlkl* or *Ripk3* significantly reduced the levels of p16, p21, TGFβ, and MMP12 in aged livers, while levels of other genes remained unaffected by the lack of *Mlkl* or *Ripk3* (Figure 4a).

**Absence of Mlkl or Ripk3 impacted non-necroptotic functions of these proteins in aged livers**

Both Mlkl and Ripk3 have several non-canonical functions that are independent of necroptosis. Wu et al reported that western diet that promotes MASH increases the expression of autophagy marker microtubule-associated protein 1 light chain 3-II (LC3-II) in the liver of mice, indicating reduced autophagic flux, and deficiency of *Mlkl* blocked this effect of western diet [24]. Therefore, we measured expressions of LC3-I and LC3-II in aged liver. Protein expression of LC3-II (1.7-fold) and its precursor LC3-I (1.26-fold) were significantly upregulated in the livers of old WT mice compared to young mice, and absence of *Ripk3*, not *Mlkl*, significantly reduced their expression (Figure 4b). Analysis of the LC3-II/LC3-I ratio revealed a significant upregulation in old WT mice (1.4-fold) compared to young mice, which was reduced in the absence of Mlkl, but not Ripk3 (Figure 4b).

Increased apoptosis of hepatocytes promotes MASH [25, 26], and Ripk3 promotes apoptosis independent of its role in necroptosis [27]. Therefore, we measured the expression of cleaved caspase-3 (CC3) in the liver, an apoptosis marker, via immunohistochemical staining. There was a 2.5-fold increase in the number of CC3-positive cells in the liver of old WT mice compared to young mice, and *Mlkl^-/-^* or *Ripk3^-/-^* old mice showed a significant reduction in liver CC3 staining (Figure 4c).

**Impact of MLKL or RIPK3 deficiency on the liver proteome in aging**

A label-free quantitative proteomic analysis of liver tissues from young WT, old WT, *Mlkl^-/-^*, and *Ripk3^-/-^* mice successfully identified and quantified a total of 2,614 unique protein groups (Table S1). A principal component analysis (PCA) of total protein abundance revealed that, despite their differences, the biological replicates in each group tightly clustered together, showing the heterogeneous nature of the liver samples (Figure 5a). Similarly, heat map clustering of the protein abundance of the total unique identified proteins further demonstrates distinct cluster among the groups (Figure 5b). Volcano plot analysis further revealed the significant (at least 1.5-fold up or down with an adjusted p-value > 0.05) difference of several proteins when compared between the groups (Figure S2 a-b). A total of 97 proteins were significantly increased, whereas 61 proteins were significantly decreased in abundance in *Mlkl^-/-^*mice compared to the aged WT mice (Figure S2a). Similarly, a total of 82 proteins were significantly increased, while 79 proteins were significantly decreased in abundance in *Ripk3^-/-^* mice compared to the old WT mice (Figure S2b). A comparison of the up- and down-regulated proteins in liver samples from the aged *Mlkl^-/-^* and *Ripk3^-/-^* with those from the aged WT mice revealed that several proteins were commonly altered between the groups (Figure 5c-d). Heat map analyses of the commonly upregulated and downregulated proteins in both *Mlkl^-/-^* and *Ripk3^-/-^* mice, relative to aged WT mice, are shown in Figures 5e and 5f. The full list of these proteins is provided in Table S1.

The dot plot in Figure 5g shows enriched Gene Ontology (GO) terms for molecular functions in the dataset. Pathway enrichment analysis revealed that the acyl-CoA hydrolase activity has the highest fold enrichment, whereas the thioester hydrolase activity pathway showed the highest statistical significance (Figure 5g). Overall, a strong enrichment of proteins involved in lipid metabolism, broad hydrolase activity, GTPase binding, and protein complex interactions were commonly upregulated in both *Mlkl^-/-^* and *Ripk3^-/-^* mice livers (Table S1). Proteins such as Acot3 and Acnat2 are associated with hydrolase activities related to acyl-CoA and CoA, suggesting a role in lipid metabolism[28]. Additionally, proteins including Acot3, Abhd12, Acnat2, and Ptrhd1 exhibit various hydrolase activities acting on ester bonds, carboxylic esters, and thiol esters, suggesting adaptive shifts in metabolic processes when necroptotic pathways are disrupted [29-32]. The presence of Diaph1, Iqgap2, Ipo9, and Afdn linked to GTPase and small GTPase binding highlights their involvement in cellular signaling and cytoskeletal organization, likely compensating for structural changes and communication needs due to necroptosis inhibition [33-36]. Furthermore, proteins such as Iqgap2, Afdn, Rnf185 and Cd74 show associations with protein complex and enzyme binding, indicating potential roles in regulating cellular processes, immune responses, and stress adaptations [37-40]. Additionally, a strong correlation exists between proteins commonly upregulated in *Mlkl^-/-^* and *Ripk3^-/-^* mice livers and the drug pregnenolone carbonitrile (Figure 5h).

**Absence of Mlkl did not impact lifespan whereas absence of Ripk3 reduced lifespan**

To determine the effect of *Mlkl or Ripk3* deficiency on overall survival, we examined the lifespan of *Mlkl^+/+^*, *Mlkl^-/-^*, *Ripk3^+/+^*, and *Ripk3^-/-^* male mice. Table 1 provides a detailed analysis of lifespan data, including mean (the average age at death for the study population), median (the age by which 50% of the population has died), and maximal lifespan (the age at death of the longest-lived individual). Mean, median, and maximum lifespan did not differ significantly between *Mlkl^+/+^* and *Mlkl^-/-^* male mice (Figure 6a, Table 1). However, mean and median lifespan of *Ripk3^-/-^* male mice was significantly lower than *Ripk3^+/+^* male mice, with no difference in their maximal lifespan (Figure 6b, Table 1). Similar results were observed for *Mlkl^-/-^* or *Ripk3^-/-^* female mice (Figure S3a-b, Table 1). Body composition analysis throughout the lifespan revealed no significant differences in body weight for male or female *Mlkl^+/+^* or *Mlkl^-/-^* mice and *Ripk3^+/+^* or *Ripk3^-/-^* (Figure 6c-d, S3c-d). Similarly, there was no significant difference in fat mass, percentage fat mass, lean mass, and percentage lean mass for male or female *Mlkl^+/+^* or *Mlkl^-/-^* mice and *Ripk3^+/+^* or *Ripk3^-/-^* mice at 22-months of age (Figure 6 e-f, S4a-d, S5a-c).

Next, we performed GTT and ITT in male mice to evaluate the effects of *Mlkl* or *Ripk3* deficiency on metabolic health in aged mice. The results indicated that aged WT and *Mlkl^-/-^* mice had similar glucose tolerance. However, aged *Ripk3^-/-^* mice displayed glucose intolerance (Figure 6g, S5d). In contrast, the ITT results showed that insulin sensitivity was comparable between WT and *Ripk3^-/-^* old mice, while aged *Mlkl^-/-^* mice exhibited improved insulin sensitivity (Figure 6h, S5e). These data suggest that Mlkl or Ripk3 deficiency exerts distinct effects on lifespan and metabolic health in aged male mice.

**Discussion**

The aim of our study was to determine the effect of absence of Mlkl or Ripk3, two key necroptosis pathway proteins, on age-related liver pathology, lifespan, and metabolic health in naturally aged mice. In our study, we targeted both Mlkl and Ripk3 due to reports of several non-canonical functions associated with these proteins [41, 42]. Therefore, if deleting Mlkl or Ripk3 have similar effects on outcome measures, we will be able to establish with relative certainty that necroptosis plays a role in driving the observed pathological processes. Our findings reveal that the genetic deletion of Mlkl or Ripk3 protects mice from age-related hepatic inflammation, steatosis, and fibrosis, hallmarks of MASLD. Notably, the absence of Mlkl or Ripk3 differently affected lifespan and metabolic health in aged mice.

Consistent with our previous report [15], we found that aging increases MLKL phosphorylation (P-MLKL), a necroptosis marker, in aged livers. An increase in necroptosis markers is reported in mouse models of diet-induced MASLD as well [12, 13, 15, 21, 43, 44]. The absence of Mlkl or Ripk3 resulted in a similar reduction of liver inflammation in aged mice, supporting the role of the Ripk3-Mlkl necroptosis pathway in liver inflammaging. In diet-induced MASLD models, the absence of MLKL consistently reduces liver inflammation [21, 24, 45], while outcomes for *Ripk3^-/-^* mice vary depending on the dietary composition [13, 24, 46]. Reduced proinflammatory macrophages in *Mlkl^-/-^* or *Ripk3^-/-^* mice suggest that necroptosis promotes a proinflammatory macrophage phenotype, contributing to chronic liver inflammation. This aligns with studies showing that necroptosis inhibition decreases proinflammatory macrophages and inflammation in the liver and other tissues [15, 21, 47-50]. As HMGB1 drives proinflammatory macrophage polarization [51, 52], the reduced circulating HMGB1 in aged *Mlkl^-/-^* or *Ripk3^-/-^* mice suggests that necroptotic HMGB1 release could influence macrophage polarization in aging. Additionally, decreased systemic proinflammatory cytokines TNFα and IL-6 in aged *Mlkl^-/-^* or *Ripk3^-/-^* mice indicate a broader anti-inflammatory effect. Consistent with our findings, Tovey Crutchfield et al. (2023) reported reduced chronic inflammation in aged *Mlkl^-/-^* mice based on inflammatory foci scoring in various tissues [53].

Aged *Mlkl^-/-^* and *Ripk3^-/-^* mice demonstrated reduced steatosis and liver fibrosis compared to their littermates, suggesting necroptosis-mediated inflammation contributes to age-related MASLD, as chronic inflammation is a known driver of MASH pathology [54-56]. Inhibition of the necroptosis pathway has been shown to reduce liver inflammation and pathology in MASH models [13, 15, 43, 57, 58]. Lower plasma ALT levels in knockout mice further indicate reduced liver injury, aligning with the protective effects of Mlkl or Ripk3 inhibition in aging liver. Consistent with previous reports showing that MLKL activation inhibits autophagy [24], absence of *Mlkl* in old mice improved LC3-II/LC3-I ratio, a marker of autophagy. However, we cannot exclude the possibility that the observed changes reflect autophagosome accumulation within lysosomes rather than altered autophagic flux [59]. Furthermore, the observed decrease in markers of apoptosis and senescence with Mlkl and Ripk3 deficiency aligns with previous studies demonstrating that RIPK3 induces apoptosis [27] and that necroptosis activation reduces senescence [15, 21]. These results indicate that both necroptosis-dependent and independent functions of Mlkl and Ripk3 influence age-related MASLD.

Our study is the first to examine the effects of Mlkl or Ripk3 deficiency on lifespan and metabolic health in 22-month-old mice, revealing distinct impacts on both. We found that at 22 months of age, *Mlkl^-/-^* mice exhibited improved insulin sensitivity whereas *Ripk3^-/-^* mice were glucose intolerant. Tovey Crutchfield et al. (2023) reported that *Mlkl^-/-^* and *Ripk3^-/-^* mice are not pre-diabetic or diabetic, however, this observation was based on blood glucose measurements in 6- and 12-month-old mice. Consistent with our findings, Rowchowdhury et al. (2016) reported glucose intolerance in 5-week-old *Ripk3^-/-^* mice on a normal chow diet [46], and we and others have shown that the absence of Mlkl improves insulin sensitivity under high-fat diet conditions [20, 60]. The different effects of Mlkl and Ripk3 deficiency on glucose and insulin tolerance in aged mice suggest their regulation of distinct metabolic pathways, independent of necroptosis. For instance, Ripk3 regulates lipid metabolism [43], while Mlkl interacts with PIP2 to influence insulin signaling [60].

While absence of Mlkl did not impact lifespan, Ripk3 deficiency reduced mean and median lifespan, however, body weights of old *Mlkl^-/-^* or *Ripk3^-/-^* mice were similar to their littermates. Li et al. (2017) reported lower body weight and youthful male reproductive organs in 15-month-old Mlkl and Ripk3 knockout mice [61]. However, a study by Tovey Crutchfield et al. (2023) reported no change in body weight or reproductive organ morphology in 12-month-old knockout mice [53]. While our findings reveal that the genetic deletion of *Mlkl* or *Ripk3* protects against age-related liver inflammation, steatosis, and fibrosis, the impact on lifespan are more complex. Although we hypothesized that reducing chronic inflammation would extend lifespan, our data indicate that the absence of *Mlkl* had no effect on lifespan, while *Ripk3* deficiency was associated with reduced mean and median lifespan. These observations challenge the expectation that ablation of necroptosis-related genes would uniformly benefit overall aging outcomes. Necroptosis is a fundamental process with adaptive roles, such as tissue regeneration [62-64], tissue homeostasis [65] and immune modulation [66, 67]. Therefore, complete elimination of this pathway could disrupt these functions, counteracting localized benefits. It is noteworthy that only few studies have shown lifespan extension from fully ablating core processes; instead, benefits often arise from modulation, as seen with growth hormone signaling [68] or caloric restriction [69]. Thus, the absence of lifespan extension in *Mlkl^-/-^* or *Ripk3^-/-^* mice, despite reduced liver inflammation and metabolic improvements, suggests that aging involves complex systemic interactions beyond the liver. Although the reasons for the reduced lifespan in *Ripk3^-/-^* mice are unclear, increased glucose intolerance with age [70] and pre-diabetic or diabetic conditions [71] are associated with reduced lifespan in humans. Thus, glucose intolerance and altered fuel utilization may negatively impact lifespan of *Ripk3^-/-^* mice.

The findings reveal that inhibiting necroptosis through Mlkl and Ripk3 knockouts drives adaptive changes in liver function, particularly enhancing lipid metabolism, cellular signaling, and immune regulation to maintain homeostasis. The strong enrichment of acyl-CoA and CoA hydrolase activities underscores the reliance on lipid turnover and energy homeostasis in the absence of necroptotic pathways. ACOX3 a peroxisomal enzyme upregulated in the absence of Mlkl or Ripk3 is responsible for the initial step of β-oxidation of branched-chain fatty acids, and caloric restriction (CR) is reported to improve lipid metabolism in the liver of mice, and ACOX3 and ACNAT2 identified in our study are the two enzymes upregulated in response to CR [72, 73]. Proteins involved in complex and enzyme binding indicate compensatory immune and stress-response mechanisms, with reductions in proinflammatory cytokines (TNFα and IL-6) further supporting an anti-inflammatory effect from necroptosis inhibition. The correlation with drugs like pregnenolone carbonitrile that exerts antifibrotic effect [74] also suggests potential pharmacological avenues to modulate similar pathways, underscoring the therapeutic promise of selective necroptosis pathway inhibition in metabolic and inflammatory liver conditions.

In conclusion, this study underscores the importance of Mlkl and Ripk3 in driving liver inflammation and MASLD in aging via their necroptotic and non-necroptotic functions. The findings suggest that targeting Mlkl or Ripk3 could provide therapeutic benefits in reducing age-related liver inflammation and pathology, however, Mlkl is a better candidate based on the adverse effects of Ripk3 deletion on glucose intolerance and lifespan. Considering that Ripk3 deletion in different cardiac cell types impacts atherosclerosis differently in *Apoe^-/-^* mice [75], our future studies will investigate the cell type- and tissue-specific effects of Mlkl in aging.

**Limitations of the study:**

The study primarily focuses on the liver, which limits the understanding of how Mlkl or Ripk3 deficiency affects other tissues and overall organismal aging. Aging is a systemic process, and effects observed in the liver may not reflect the impacts on other critical tissues. The study does not provide insights into how Mlkl or Ripk3 deficiency affects systemic aging processes or interactions between different tissues. Additionally, detailed necropsy or pathological analyses at the time of death were not performed in this study. This lack of data limits our ability to identify the underlying causes of the shorter lifespan observed in *Ripk3^-^*^/-^ mice. Addressing these limitations in future research could provide a more comprehensive understanding of how Mlkl or Ripk3 impact aging and lifespan.

**Data Availability Statement**

Proteomics data can be found in the MassIVE database via MSV000096299. Additional data supporting the study’s findings are provided within the manuscript and its supplementary materials. Correspondence and requests for information should be addressed to S. Deepa.

**Acknowledgements**

N. Ahsan gratefully acknowledges the initial funding support from the OU VPRP Office for the establishment of the Proteomics Core Facility. The authors thank the Stephenson Cancer Tissue Pathology Core for the histology and immunohistochemistry services provided which was supported by the National Institute of General Medical Sciences Grant P20GM103639 and National Cancer Institute Grant P30CA225520 of the National Institutes of Health. We thank Dr. Arlan Richardson (Professor, University of Oklahoma Health Sciences Center, and Oklahoma City Veterans Affairs Medical Center) for generously sharing the liver tissue from mice that were generated and housed at the Oklahoma City Veterans Affairs Health Care System Animal Facility.

**Funding**

This work was supported by NIH grants R01AG059718 and R03CA262044 to S. Deepa.

**Author Contributions**

S. Mohammed performed and analyzed experiments, prepared figures, manuscript writing; P. Ohene-Marfo, C. Jiang manuscript writing and editing; Z. Peng did the LC-MS/MS analysis and proteomics data analysis; N. Thadathil helped with sample collection and performed experiments; A. Tran, E. Nicklas, D. Wang, R. Selvarani helped with the animal studies; A. Singh. and Z. Yang did the proteomic data analysis; S. Bhaskaran gave critical comments and suggestion for the manuscript; N. Ahsan did the LC-MS/MS analysis, proteomics data analysis, writing, review, and editing of the manuscript; S. Deepa designed the experiments, supervised the research, writing, review, and editing of the manuscript.

**Ethical Declarations**

**Ethical Approval**

This study was approved by the Institutional Animal Care and Use Committee at the University of Oklahoma Health Sciences Center (OUHSC); protocol numbers 18-112-EA and 21-074-EAFHI.

**Consent to participate**

The study did not involve human subjects.

**Consent to publish**

All authors have reviewed the content of this research and approved it for publication.

**Competing Interests**

The authors declare no competing financial interests.

**References**

1. Franceschi C, Campisi J. Chronic inflammation (inflammaging) and its potential contribution to age-associated diseases. J Gerontol A Biol Sci Med Sci. 2014;69 Suppl 1:S4-9. doi: 10.1093/gerona/glu057.

2. Kennedy BK, Berger SL, Brunet A, Campisi J, Cuervo AM, Epel ES, et al. Geroscience: linking aging to chronic disease. Cell. 2014;159(4):709-13. doi: 10.1016/j.cell.2014.10.039.

3. Ferrucci L, Fabbri E. Inflammageing: chronic inflammation in ageing, cardiovascular disease, and frailty. Nat Rev Cardiol. 2018;15(9):505-22. doi: 10.1038/s41569-018-0064-2.

4. Jaul E, Barron J. Age-Related Diseases and Clinical and Public Health Implications for the 85 Years Old and Over Population. Front Public Health. 2017;5:335. doi: 10.3389/fpubh.2017.00335.

5. Kesidou E, Theotokis P, Damianidou O, Boziki M, Konstantinidou N, Taloumtzis C, et al. CNS Ageing in Health and Neurodegenerative Disorders. J Clin Med. 2023;12(6). doi: 10.3390/jcm12062255.

6. Golabi P, Paik J, Reddy R, Bugianesi E, Trimble G, Younossi ZM. Prevalence and long-term outcomes of non-alcoholic fatty liver disease among elderly individuals from the United States. BMC Gastroenterol. 2019;19(1):56. doi: 10.1186/s12876-019-0972-6.

7. Younossi ZM. Non-alcoholic fatty liver disease - A global public health perspective. J Hepatol. 2019;70(3):531-44. doi: 10.1016/j.jhep.2018.10.033.

8. Younossi Z, Stepanova M, Ong JP, Jacobson IM, Bugianesi E, Duseja A, et al. Nonalcoholic Steatohepatitis Is the Fastest Growing Cause of Hepatocellular Carcinoma in Liver Transplant Candidates. Clin Gastroenterol Hepatol. 2019;17(4):748-55.e3. doi: 10.1016/j.cgh.2018.05.057.

9. Pais R, Redheuil A, Cluzel P, Ratziu V, Giral P. Relationship Among Fatty Liver, Specific and Multiple-Site Atherosclerosis, and 10-Year Framingham Score. Hepatology. 2019;69(4):1453-63. doi: 10.1002/hep.30223.

10. Stepanova M, Kabbara K, Mohess D, Verma M, Roche-Green A, AlQahtani S, et al. Nonalcoholic steatohepatitis is the most common indication for liver transplantation among the elderly: Data from the United States Scientific Registry of Transplant Recipients. Hepatol Commun. 2022;6(7):1506-15. doi: 10.1002/hep4.1915.

11. Harrison SA, Bedossa P, Guy CD, Schattenberg JM, Loomba R, Taub R, et al. A Phase 3, Randomized, Controlled Trial of Resmetirom in NASH with Liver Fibrosis. N Engl J Med. 2024;390(6):497-509. doi: 10.1056/NEJMoa2309000.

12. Miyata T, Wu X, Fan X, Huang E, Sanz-Garcia C, Ross CKC, et al. Differential role of MLKL in alcohol-associated and non-alcohol-associated fatty liver diseases in mice and humans. JCI Insight. 2021;6(4). doi: 10.1172/jci.insight.140180.

13. Gautheron J, Vucur M, Reisinger F, Cardenas DV, Roderburg C, Koppe C, et al. A positive feedback loop between RIP3 and JNK controls non-alcoholic steatohepatitis. EMBO Mol Med. 2014;6(8):1062-74. doi: 10.15252/emmm.201403856.

14. van Loo G, Bertrand MJM. Death by TNF: a road to inflammation. Nat Rev Immunol. 2023;23(5):289-303. doi: 10.1038/s41577-022-00792-3.

15. Mohammed S, Thadathil N, Selvarani R, Nicklas EH, Wang D, Miller BF, et al. Necroptosis contributes to chronic inflammation and fibrosis in aging liver. Aging Cell. 2021;20(12):e13512. doi: 10.1111/acel.13512.

16. Mohammed S, Nicklas EH, Thadathil N, Selvarani R, Royce GH, Kinter M, et al. Role of necroptosis in chronic hepatic inflammation and fibrosis in a mouse model of increased oxidative stress. Free Radic Biol Med. 2021;164:315-28. doi: 10.1016/j.freeradbiomed.2020.12.449.

17. Royce GH, Brown-Borg HM, Deepa SS. The potential role of necroptosis in inflammaging and aging. Geroscience. 2019;41(6):795-811. doi: 10.1007/s11357-019-00131-w.

18. Newton K, Sun X, Dixit VM. Kinase RIP3 is dispensable for normal NF-kappa Bs, signaling by the B-cell and T-cell receptors, tumor necrosis factor receptor 1, and Toll-like receptors 2 and 4. Mol Cell Biol. 2004;24(4):1464-9. doi: 10.1128/mcb.24.4.1464-1469.2004.

19. Murphy JM, Czabotar PE, Hildebrand JM, Lucet IS, Zhang JG, Alvarez-Diaz S, et al. The pseudokinase MLKL mediates necroptosis via a molecular switch mechanism. Immunity. 2013;39(3):443-53. doi: 10.1016/j.immuni.2013.06.018.

20. Ohene-Marfo P, Nguyen HVM, Mohammed S, Thadathil N, Tran A, Nicklas EH, et al. Non-Necroptotic Roles of MLKL in Diet-Induced Obesity, Liver Pathology, and Insulin Sensitivity: Insights from a High-Fat, High-Fructose, High-Cholesterol Diet Mouse Model. Int J Mol Sci. 2024;25(5). doi: 10.3390/ijms25052813.

21. Mohammed S, Thadathil N, Ohene-Marfo P, Tran AL, Van Der Veldt M, Georgescu C, et al. Absence of Either Ripk3 or Mlkl Reduces Incidence of Hepatocellular Carcinoma Independent of Liver Fibrosis. Mol Cancer Res. 2023;21(9):933-46. doi: 10.1158/1541-7786.Mcr-22-0820.

22. Ahsan N, Fornelli L, Najar FZ, Gamagedara S, Hossan MR, Rao RSP, et al. Proteomics evaluation of five economical commercial abundant protein depletion kits for enrichment of diseases-specific biomarkers from blood serum. Proteomics. 2023;23(20):e2300150. doi: 10.1002/pmic.202300150.

23. Ge SX, Jung D, Yao R. ShinyGO: a graphical gene-set enrichment tool for animals and plants. Bioinformatics. 2020;36(8):2628-9. doi: 10.1093/bioinformatics/btz931.

24. Wu X, Poulsen KL, Sanz-Garcia C, Huang E, McMullen MR, Roychowdhury S, et al. MLKL-dependent signaling regulates autophagic flux in a murine model of non-alcohol-associated fatty liver and steatohepatitis. J Hepatol. 2020;73(3):616-27. doi: 10.1016/j.jhep.2020.03.023.

25. Feldstein AE, Canbay A, Angulo P, Taniai M, Burgart LJ, Lindor KD, et al. Hepatocyte apoptosis and fas expression are prominent features of human nonalcoholic steatohepatitis. Gastroenterology. 2003;125(2):437-43. doi: 10.1016/s0016-5085(03)00907-7.

26. Thapaliya S, Wree A, Povero D, Inzaugarat ME, Berk M, Dixon L, et al. Caspase 3 inactivation protects against hepatic cell death and ameliorates fibrogenesis in a diet-induced NASH model. Dig Dis Sci. 2014;59(6):1197-206. doi: 10.1007/s10620-014-3167-6.

27. Mandal P, Berger SB, Pillay S, Moriwaki K, Huang C, Guo H, et al. RIP3 induces apoptosis independent of pronecrotic kinase activity. Mol Cell. 2014;56(4):481-95. doi: 10.1016/j.molcel.2014.10.021.

28. Hunt MC, Siponen MI, Alexson SE. The emerging role of acyl-CoA thioesterases and acyltransferases in regulating peroxisomal lipid metabolism. Biochim Biophys Acta. 2012;1822(9):1397-410. doi: 10.1016/j.bbadis.2012.03.009.

29. Hunt MC, Rautanen A, Westin MA, Svensson LT, Alexson SE. Analysis of the mouse and human acyl-CoA thioesterase (ACOT) gene clusters shows that convergent, functional evolution results in a reduced number of human peroxisomal ACOTs. Faseb j. 2006;20(11):1855-64. doi: 10.1096/fj.06-6042com.

30. Joshi A, Shaikh M, Singh S, Rajendran A, Mhetre A, Kamat SS. Biochemical characterization of the PHARC-associated serine hydrolase ABHD12 reveals its preference for very-long-chain lipids. J Biol Chem. 2018;293(44):16953-63. doi: 10.1074/jbc.RA118.005640.

31. Hunt MC, Alexson SE. Novel functions of acyl-CoA thioesterases and acyltransferases as auxiliary enzymes in peroxisomal lipid metabolism. Prog Lipid Res. 2008;47(6):405-21. doi: 10.1016/j.plipres.2008.05.001.

32. Taylor-Creel K, Hames MC, Holloway WB, McFeeters H, McFeeters RL. Expression, purification, and solubility optimization of peptidyl-tRNA hydrolase 1 from Bacillus cereus. Protein Expr Purif. 2014;95:259-64. doi: 10.1016/j.pep.2014.01.007.

33. Li D, Dammer EB, Lucki NC, Sewer MB. cAMP-stimulated phosphorylation of diaphanous 1 regulates protein stability and interaction with binding partners in adrenocortical cells. Mol Biol Cell. 2013;24(6):848-57. doi: 10.1091/mbc.E12-08-0597.

34. Schmidt VA, Scudder L, Devoe CE, Bernards A, Cupit LD, Bahou WF. IQGAP2 functions as a GTP-dependent effector protein in thrombin-induced platelet cytoskeletal reorganization. Blood. 2003;101(8):3021-8. doi: 10.1182/blood-2002-09-2807.

35. Dopie J, Skarp KP, Rajakylä EK, Tanhuanpää K, Vartiainen MK. Active maintenance of nuclear actin by importin 9 supports transcription. Proc Natl Acad Sci U S A. 2012;109(9):E544-52. doi: 10.1073/pnas.1118880109.

36. Goudreault M, Gagné V, Jo CH, Singh S, Killoran RC, Gingras AC, et al. Afadin couples RAS GTPases to the polarity rheostat Scribble. Nat Commun. 2022;13(1):4562. doi: 10.1038/s41467-022-32335-8.

37. Song F, Dai Q, Grimm MO, Steinbach D. The Antithetic Roles of IQGAP2 and IQGAP3 in Cancers. Cancers (Basel). 2023;15(4). doi: 10.3390/cancers15041115.

38. van de Weijer ML, Samanta K, Sergejevs N, Jiang L, Dueñas ME, Heunis T, et al. Tapasin assembly surveillance by the RNF185/Membralin ubiquitin ligase complex regulates MHC-I surface expression. Nat Commun. 2024;15(1):8508. doi: 10.1038/s41467-024-52772-x.

39. Zhang L, Woltering I, Holzner M, Brandhofer M, Schaefer CC, Bushati G, et al. CD74 is a functional MIF receptor on activated CD4(+) T cells. Cell Mol Life Sci. 2024;81(1):296. doi: 10.1007/s00018-024-05338-5.

40. Bonucci M, Kuperwasser N, Barbe S, Koka V, de Villeneuve D, Zhang C, et al. mTOR and S6K1 drive polycystic kidney by the control of Afadin-dependent oriented cell division. Nat Commun. 2020;11(1):3200. doi: 10.1038/s41467-020-16978-z.

41. Zhan C, Huang M, Yang X, Hou J. MLKL: Functions beyond serving as the Executioner of Necroptosis. Theranostics. 2021;11(10):4759-69. doi: 10.7150/thno.54072.

42. Zhou Y, Xiang Y, Liu S, Li C, Dong J, Kong X, et al. RIPK3 signaling and its role in regulated cell death and diseases. Cell Death Discov. 2024;10(1):200. doi: 10.1038/s41420-024-01957-w.

43. Afonso MB, Rodrigues PM, Mateus-Pinheiro M, Simão AL, Gaspar MM, Majdi A, et al. RIPK3 acts as a lipid metabolism regulator contributing to inflammation and carcinogenesis in non-alcoholic fatty liver disease. Gut. 2021;70(12):2359-72. doi: 10.1136/gutjnl-2020-321767.

44. Afonso MB, Rodrigues PM, Carvalho T, Caridade M, Borralho P, Cortez-Pinto H, et al. Necroptosis is a key pathogenic event in human and experimental murine models of non-alcoholic steatohepatitis. Clin Sci (Lond). 2015;129(8):721-39. doi: 10.1042/cs20140732.

45. Wu X, Nagy LE. MLKL contributes to Western diet-induced liver injury through inhibiting autophagy. Autophagy. 2020;16(7):1351-2. doi: 10.1080/15548627.2020.1760624.

46. Roychowdhury S, McCullough RL, Sanz-Garcia C, Saikia P, Alkhouri N, Matloob A, et al. Receptor interacting protein 3 protects mice from high-fat diet-induced liver injury. Hepatology. 2016;64(5):1518-33. doi: 10.1002/hep.28676.

47. Xu J, Wu D, Zhou S, Hu H, Li F, Guan Z, et al. MLKL deficiency attenuated hepatocyte oxidative DNA damage by activating mitophagy to suppress macrophage cGAS-STING signaling during liver ischemia and reperfusion injury. Cell Death Discov. 2023;9(1):58. doi: 10.1038/s41420-023-01357-6.

48. Kitur K, Parker D, Nieto P, Ahn DS, Cohen TS, Chung S, et al. Toxin-induced necroptosis is a major mechanism of Staphylococcus aureus lung damage. PLoS Pathog. 2015;11(4):e1004820. doi: 10.1371/journal.ppat.1004820.

49. Yang J, Zhao Y, Zhang L, Fan H, Qi C, Zhang K, et al. RIPK3/MLKL-Mediated Neuronal Necroptosis Modulates the M1/M2 Polarization of Microglia/Macrophages in the Ischemic Cortex. Cereb Cortex. 2018;28(7):2622-35. doi: 10.1093/cercor/bhy089.

50. Chen H, Fang Y, Wu J, Chen H, Zou Z, Zhang X, et al. RIPK3-MLKL-mediated necroinflammation contributes to AKI progression to CKD. Cell Death Dis. 2018;9(9):878. doi: 10.1038/s41419-018-0936-8.

51. He C, Sun S, Zhang Y, Xie F, Li S. The role of irreversible electroporation in promoting M1 macrophage polarization via regulating the HMGB1-RAGE-MAPK axis in pancreatic cancer. Oncoimmunology. 2021;10(1):1897295. doi: 10.1080/2162402x.2021.1897295.

52. Kigerl KA, Lai W, Wallace LM, Yang H, Popovich PG. High mobility group box-1 (HMGB1) is increased in injured mouse spinal cord and can elicit neurotoxic inflammation. Brain Behav Immun. 2018;72:22-33. doi: 10.1016/j.bbi.2017.11.018.

53. Tovey Crutchfield EC, Garnish SE, Day J, Anderton H, Chiou S, Hempel A, et al. MLKL deficiency protects against low-grade, sterile inflammation in aged mice. Cell Death Differ. 2023;30(4):1059-71. doi: 10.1038/s41418-023-01121-4.

54. Li Z, Yang S, Lin H, Huang J, Watkins PA, Moser AB, et al. Probiotics and antibodies to TNF inhibit inflammatory activity and improve nonalcoholic fatty liver disease. Hepatology. 2003;37(2):343-50. doi: 10.1053/jhep.2003.50048.

55. Klover PJ, Clementi AH, Mooney RA. Interleukin-6 depletion selectively improves hepatic insulin action in obesity. Endocrinology. 2005;146(8):3417-27. doi: 10.1210/en.2004-1468.

56. Tanwar S, Rhodes F, Srivastava A, Trembling PM, Rosenberg WM. Inflammation and fibrosis in chronic liver diseases including non-alcoholic fatty liver disease and hepatitis C. World J Gastroenterol. 2020;26(2):109-33. doi: 10.3748/wjg.v26.i2.109.

57. Miyata T, Wu X, Fan X, Huang E, Sanz-Garcia C, Ross CKC, et al. Differential role of MLKL in alcohol-associated and non-alcohol-associated fatty liver diseases in mice and humans. JCI Insight. 2022;7(23). doi: 10.1172/jci.insight.167011.

58. Guo R, Jia X, Ding Z, Wang G, Jiang M, Li B, et al. Loss of MLKL ameliorates liver fibrosis by inhibiting hepatocyte necroptosis and hepatic stellate cell activation. Theranostics. 2022;12(11):5220-36. doi: 10.7150/thno.71400.

59. Ueno T, Komatsu M. Monitoring Autophagy Flux and Activity: Principles and Applications. Bioessays. 2020;42(11):e2000122. doi: 10.1002/bies.202000122.

60. Xu H, Du X, Liu G, Huang S, Du W, Zou S, et al. The pseudokinase MLKL regulates hepatic insulin sensitivity independently of inflammation. Mol Metab. 2019;23:14-23. doi: 10.1016/j.molmet.2019.02.003.

61. Li D, Meng L, Xu T, Su Y, Liu X, Zhang Z, et al. RIPK1-RIPK3-MLKL-dependent necrosis promotes the aging of mouse male reproductive system. Elife. 2017;6. doi: 10.7554/eLife.27692.

62. Zhou S, Zhang W, Cai G, Ding Y, Wei C, Li S, et al. Myofiber necroptosis promotes muscle stem cell proliferation via releasing Tenascin-C during regeneration. Cell Res. 2020;30(12):1063-77. doi: 10.1038/s41422-020-00393-6.

63. Lloyd AF, Davies CL, Holloway RK, Labrak Y, Ireland G, Carradori D, et al. Central nervous system regeneration is driven by microglia necroptosis and repopulation. Nat Neurosci. 2019;22(7):1046-52. doi: 10.1038/s41593-019-0418-z.

64. Ying Z, Pan C, Shao T, Liu L, Li L, Guo D, et al. Mixed Lineage Kinase Domain-like Protein MLKL Breaks Down Myelin following Nerve Injury. Mol Cell. 2018;72(3):457-68.e5. doi: 10.1016/j.molcel.2018.09.011.

65. Yin J, Yu Y, Huang X, Chan FK. Necroptosis in immunity, tissue homeostasis, and cancer. Curr Opin Immunol. 2024;89:102455. doi: 10.1016/j.coi.2024.102455.

66. Nailwal H, Chan FK. Necroptosis in anti-viral inflammation. Cell Death Differ. 2019;26(1):4-13. doi: 10.1038/s41418-018-0172-x.

67. Lu JV, Chen HC, Walsh CM. Necroptotic signaling in adaptive and innate immunity. Semin Cell Dev Biol. 2014;35:33-9. doi: 10.1016/j.semcdb.2014.07.003.

68. Aguiar-Oliveira MH, Bartke A. Growth Hormone Deficiency: Health and Longevity. Endocr Rev. 2019;40(2):575-601. doi: 10.1210/er.2018-00216.

69. Anderson RM, Shanmuganayagam D, Weindruch R. Caloric restriction and aging: studies in mice and monkeys. Toxicol Pathol. 2009;37(1):47-51. doi: 10.1177/0192623308329476.

70. Kalyani RR, Egan JM. Diabetes and altered glucose metabolism with aging. Endocrinol Metab Clin North Am. 2013;42(2):333-47. doi: 10.1016/j.ecl.2013.02.010.

71. Rapoport M, Chetrit A, Cantrell D, Novikov I, Roth J, Dankner R. Years of potential life lost in pre-diabetes and diabetes mellitus: data from a 40-year follow-up of the Israel study on Glucose intolerance, Obesity and Hypertension. BMJ Open Diabetes Res Care. 2021;9(1). doi: 10.1136/bmjdrc-2020-001981.

72. Mezhnina V, Pearce R, Poe A, Velingkaar N, Astafev A, Ebeigbe OP, et al. CR reprograms acetyl-CoA metabolism and induces long-chain acyl-CoA dehydrogenase and CrAT expression. Aging Cell. 2020;19(11):e13266. doi: 10.1111/acel.13266.

73. Makwana K, Gosai N, Poe A, Kondratov RV. Calorie restriction reprograms diurnal rhythms in protein translation to regulate metabolism. Faseb j. 2019;33(3):4473-89. doi: 10.1096/fj.201802167R.

74. Marek CJ, Tucker SJ, Konstantinou DK, Elrick LJ, Haefner D, Sigalas C, et al. Pregnenolone-16alpha-carbonitrile inhibits rodent liver fibrogenesis via PXR (pregnane X receptor)-dependent and PXR-independent mechanisms. Biochem J. 2005;387(Pt 3):601-8. doi: 10.1042/bj20041598.

75. Colijn S, Muthukumar V, Xie J, Gao S, Griffin CT. Cell-specific and athero-protective roles for RIPK3 in a murine model of atherosclerosis. Dis Model Mech. 2020;13(1). doi: 10.1242/dmm.041962.

**Figure Legends**

**Fig. 1** Markers of necroptosis in young and old livers. (a) Gross liver weight in grams (on the left) and percentage liver weight (normalized to body mass (BM) (on the right) of young (7-month-old) WT or old WT, *Mlkl^−/−^* or *Ripk3^−/−^* (24-month-old) male mice (b) Top: Immunoblots of liver tissue extracts for necroptosis proteins: Mlkl, Ripk3 and β-actin. Bottom: Graphical representation of quantified blot normalized to β-actin. (c) Transcript levels of *Mlkl* and *Ripk3* (d) *Left*: Representative IHC staining for P-MLKL in liver sections. *Right*: Graphical representation of the intensity of staining. Scale bar: 50 μm. (e) Levels of HMGB1 in circulation. White, red, yellow and blue bars represent young WT, old WT, old *Mlkl^−/−^* and old *Ripk3^−/−^* respectively (n=4-6 group). Error bars are represented as mean ± SEM. One-way ANOVA, * p< 0.05, ** p< 0.005, *** p<0.0005. ANOVA summary (F value, p value): (a, left) 1.2, 0.34; (a, right) 4.83, 0.01; (b, left) 364.6, <0.0001; (b, right) 9.04, 0.002; (c, left) 43.6, <0.0001; (c, right) 17.88, <0.0001; (d) 19.37, <0.0001; (e) 7.48, 0.002.

**Fig. 2** Absence of Mlkl and Ripk3 reduces hepatic inflammation. (a) *Left*: Representative images for IHC staining for F4/80 (brown) counterstained with hematoxylin (purple) in liver sections of experimental mice. *Right*: Graphical representation of number of F4/80 positive cells detected per microscopic field. Scale bar: 100 μm. Transcript levels (b) *CD11c*, *CD86* and *CD 68, Arg1* and *Fizz1* (c) *TNFα, IL6, IL-1β* and *MCP1* normalized with respect to housekeeping genes and represented as fold change relative to young WT group (d) Circulating levels of TNFα, and IL6 (n=6-8/group). White, red, yellow and blue bars represent young WT, old WT, old *Mlkl^−/−^* and old *Ripk3^−/−^* respectively (Figures a-c: n=4-6 group). Error bars are represented as mean ± SEM. One-way ANOVA, * p< 0.05, ** p< 0.005, *** p<0.0005. ANOVA summary (F value, p value): (a) 18.3, <0.0001; (b , *CD11c*) 6.85, 0.005; (b, *CD86*) 5.6, 0.01; (b, *CD68*) 8.8, 0.002; (b, *Arg1*) 9.45, 0.002; (b, *Fizz1*) 5.25, 0.014; (c, *TNFα*) 7.73, 0.003; (c, *IL6*) 8.95, 0.002; (c, *IL-1β*) 10.2, 0.001; (c, *MCP1*) 10.5, 0.0009; (d, *TNFα*) 8.42, 0.0005; (d, IL6) 24.26, <0.0001

**Fig. 3** Absence of Mlkl and Ripk3 protects from age related liver pathology. (a) *Left*: Representative images of H&E-stained liver sections. Scale bar: 100μM; *Right*: Graphical representation of steatosis in the livers of experimental mice groups (b) Quantification of total triglyceride in liver tissue (c) *Left:* Representative images of PSR staining of liver sections. Scale bar: 100μM. *Right:* Quantification of PSR staining, represented as percentage area. (d) The transcript levels of fibrosis markers normalized with respect to housekeeping genes and represented as fold change relative to young WT group (e) Levels of ALT in plasma (n=6-8 group). White, red, yellow and blue bars represent young WT, old WT, old *Mlkl^−/−^* or old *Ripk3^−/−^* respectively (Figures a-d: n=4-6 group). Error bars are represented as mean ± SEM. One-way ANOVA, * p< 0.05, ** p< 0.005, *** p<0.0005. ANOVA summary (F value, p value): (a) 39.74, <0.0001; (b) 10.14, 0.0006; (c) 50.6, <0.0001; (d, *Acta2*) 9.59, 0.001; (d, *Col1α1*) 11.97, 0.0004; (e) 18.66, <0.0001

**Fig. 4** Effect of absence of Mlkl and Ripk3 on non-canonical functions in aged liver. (a) The transcript levels of *p16,* *p21*, *p19*, *TGFβ*, *MMP12*, *MMP3* and *CXCL2* normalized with respect to housekeeping genes and represented as fold change relative to young WT group. (b) *Left*: Immunoblots of liver tissue extracts for LC3-I, LC3-II (autophagy markers) and β-actin. *Right*: Graphical representation of quantified immunoblot normalized to β-actin and ratio of LC3-II/I. *Left:* Representative images for IHC staining for cleaved caspase-3 in liver sections. *Right:* Graphical representation of number of cleaved caspase-3 positive cells (arrow heads) per microscopic field. Scale bar: 100μm. White, red, yellow and blue bars represent young WT, old WT, old *Mlkl^−/−^* or old *Ripk3^−/−^* respectively (n= 4-6/ group). Error bars are represented as mean ± SEM. One-way ANOVA, * p< 0.05, ** p< 0.005, *** p<0.0005. ANOVA summary (F value, p value): (a, *p16*) 12.49, 0.0002; (a, *p21*) 9.34, 0.0008; (a, *p19*) 12.59, 0.0002; (a, *TGFβ*) 20.46, <0.0001; (a, *MMP12*) 11.57, 0.0002; (a, MMP3) 8.62, 0.0007; (a, CXCL2) 11.15, 0.0002; (b, LC3-I) 7.88, 0.0036; (b, LC3-II) 12.19, 0.0006; (b, LC3-II/LC3-I) 5.73, 0.01; (c) 11.64, <0.0001

**Fig. 5** Effect of absence of Mlkl and Ripk3 on aging liver proteome. (a) Principal Component Analysis (PCA) score plot of proteomics data from livers of young WT (white circles), old WT (red circles), old *Mlkl^−/−^* (yellow circles) or old *Ripk3^−/−^* (blue circles) mice. (n= 5 per group) (b) Heat map clustering of the protein abundance of the total unique identified proteins in the experimental groups. Venn diagram of the number of commonly (c) upregulated and (d) downregulated proteins in the livers of old *Mlkl^−/−^* and old *Ripk3^−/−^* mice. Heat map analyses of the commonly (e) upregulated and (f) downregulated proteins in the livers of old *Mlkl^−/−^* and old *Ripk3^−/−^* mice. (g) Gene Ontology (GO) molecular pathway analysis of the commonly upregulated proteins in (c) and (e). (h) Drug Comparative Toxicogenomics Database (Drug.CTD) enrichment bubble analysis of the commonly upregulated proteins in (c) and (e)

**Fig 6** Effect of absence of *Ripk3 or Mlkl* on survival, body weight and body composition of male mice. Kaplan-Meier survival curves for (a) *Mlkl^+/+^, Mlkl^-/-^* (on the left) mice or (b) *Ripk3^+/+^, Ripk3^-/-^* mice (on the right). Average body weight changes of (c) *Mlkl^+/+^*, *Mlkl^-/-^* and (d) *Ripk3^+/+^, Ripk3^-/-^* mice. Percentage of fat mass normalized to total body weight of (e) *Mlkl^+/+^*, *Mlkl^-/-^* and (f) *Ripk3^+/+^, Ripk3^-/-^* mice. (g) Glucose Tolerance Test (GTT) (h) Insulin Tolerance Test (ITT) of old WT, old *Mlkl^-/-^* and old *Ripk3^-/-^* mice (all three groups were 22 months of age). GTT and ITT was performed with the same mice, with a time gap of 10 days between the tests. a, b: n= 42 *Mlkl^+/+^*, n=45 *Mlkl^-/-^,* n=40 *Ripk3^+/+^*, n=42 *Ripk3^-/-^*. c-h: n=10/ group. Data are expressed as mean ± SEM and analyzed using two-way ANOVA. * p<0.05. For g, h: #: Represents significant difference between old WT vs old *Mlkl^-/-^*, &: old WT vs old *Ripk3^-/-^*, @: old *Mlkl^-/-^* vs old *Ripk3^-/-^*. ANOVA summary (F value, p value): (c) row factor (time points) 36.24, <0.0001 column factor (genotype) 1.28, 0.26; (d) row factor (time points) 22.56, <0.0001 column factor (genotype) 0.052, 0.82; (e) row factor (time points) 2.61, 0.021 column factor (genotype) 2.64, 0.107; (f) row factor (time points) 11.08, <0.0001 column factor (genotype) 3.73, 0.056; (g) row factor (time points) 57.49, <0.0001 column factor (genotype) 26.55, <0.0001; (h) row factor (time points) 22.48, <0.0001 column factor (genotype) 12.74, <0.0001

| **MALES** | | |
| --- | --- | --- |
|  | ***Mlkl^+/+^*** | ***Mlkl^-/-^*** |
| Mean + SEM | 806.6+38^@^ | 769.2+41.32 |
| Median | 847.5 | 853.0 |
| Maximum | 1204 | 1122 |
| 10% Percentile | 488.1 | 304.0 |
| 25% Percentile | 616.0 | 584.5 |
| 75% Percentile | 981.3 | 1016 |
| 90% percentile | 1137 | 1054 |
| Number of mice | 42 | 45 |
|  | ***Ripk3^+/+^*** | ***Ripk3^-/-^*** |
| Mean + SEM | 930.45+22.65* | 838.6+28.74 |
| Median | 976.5* | 885 |
| Maximum | 1114 | 1103 |
| 10% Percentile | 695.5 | 578.2 |
| 25% Percentile | 889.5 | 734.3 |
| 75% Percentile | 1014 | 959.5 |
| 90% percentile | 1090 | 1024 |
| Number of mice | 40 | 42 |
| **FEMALES** | | |
|  | ***Mlkl^+/+^*** | ***Mlkl^-/-^*** |
| Mean + SEM | 786.4+27.73 | 834.9+26.13^#^ |
| Median | 839 | 911 |
| Maximum | 1068 | 1056 |
| 10% Percentile | 448.5 | 536.6 |
| 25% Percentile | 728.8 | 729.5 |
| 75% Percentile | 907.0 | 957.5 |
| 90% percentile | 998.5 | 990.8 |
| Number of mice | 44 | 45 |
|  | ***Ripk3^+/+^*** | ***Ripk3^-/-^*** |
| Mean + SEM | 839+26.84* | 742.8+34.11 |
| Median | 889.0 | 872.0 |
| Maximum | 1104 | 1029 |
| 10% Percentile | 521.4 | 351.5 |
| 25% Percentile | 793.5 | 562.5 |
| 75% Percentile | 949.0 | 914.5 |
| 90% percentile | 1004 | 962.5 |
| Number of mice | 45 | 44 |

**Table 1**: Lifespan analysis of male and female *Mlkl^+/+^* and *Mlkl^-/-^* , *Ripk3^+/+^* and *Ripk3^-/-^* mice.

The survival data from Figure 6 and Figure S2 are expressed in days. * Indicates significant difference between genotypes of same sex. @ indicates significant difference between male *Mlkl^+/+^* and *Ripk3^+/+^* mice. # indicates significant difference between female *Mlkl^-/-^* and *Ripk3^-/-^* mice.

**Supplementary information: GeroScience**

**Impact of Mlkl or Ripk3 deletion on age-associated liver inflammation, metabolic health, and lifespan**

Sabira Mohammed^1,2^, Phoebe Ohene-Marfo^2^, Chao Jiang^1,2^, Zongkai Peng^3^, Nidheesh Thadathil^2^, Albert Tran^2^, Evan Nicklas^2^, Shylesh Bhaskaran^1,2^, Dawei Wang^2^, Ramasamy Selvarani^2^, Amit Sing^3^, Zhibo Yang^1,3^, Nagib Ahsan^3,4^, Sathyaseelan S. Deepa^1,2,5^*

1Stephenson Cancer Center, University of Oklahoma Health Sciences Center, Oklahoma City, Oklahoma, USA

2Department of Biochemistry & Physiology, University of Oklahoma Health Sciences Center, Oklahoma City, Oklahoma, USA

3 Department of Chemistry and Biochemistry, University of Oklahoma, Norman, OK, USA

4 Mass Spectrometry, Proteomics and Metabolomics Core Facility, Stephenson Life Sciences Research Center, The University of Oklahoma, Norman, OK, USA

5Oklahoma Center for Geroscience & Brain Aging, University of Oklahoma Health Sciences Center, Oklahoma City, Oklahoma, USA

Corresponding Author: Sathyaseelan S Deepa, Ph.D.

Stephenson Cancer Center

Department of Biochemistry and Physiology

Oklahoma Center for Geroscience & Brain Aging

University of Oklahoma Health Sciences Center

975 NE 10th Street, BRC-1368A

Oklahoma City, OK 73104, USA

E-mail: [Deepa-Sathyaseelan@ouhsc.edu](mailto:Deepa-Sathyaseelan@ouhsc.edu)


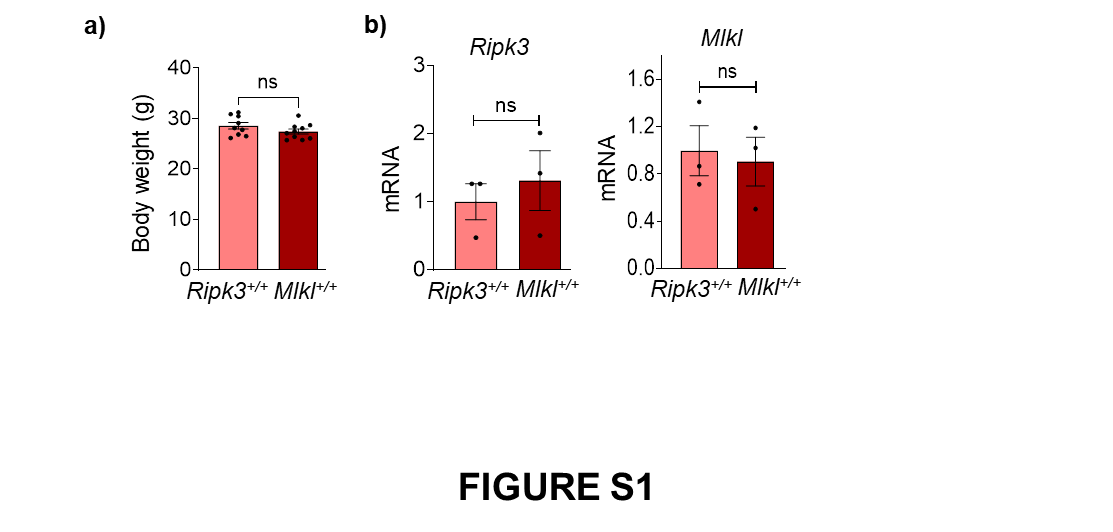


**Fig. S1 (**a) Body weight of *Ripk3^+/+^* and *Mlkl^+/+^* mice used for the study measured at 3 months of age (b) Transcript levels of *Mlkl* and *Ripk3* in the livers of *Ripk3^+/+^* and *Mlkl^+/+^* mice measured at 24 months of age. Error bars are represented as mean ± SEM. Unpaired t-test, ns: p>0.05. (F value, p value): (a) 1.55, 0.522; (b, *Mlkl*) 1.05, 0.97; (b, *Ripk3*) 2.76, 0.53

**
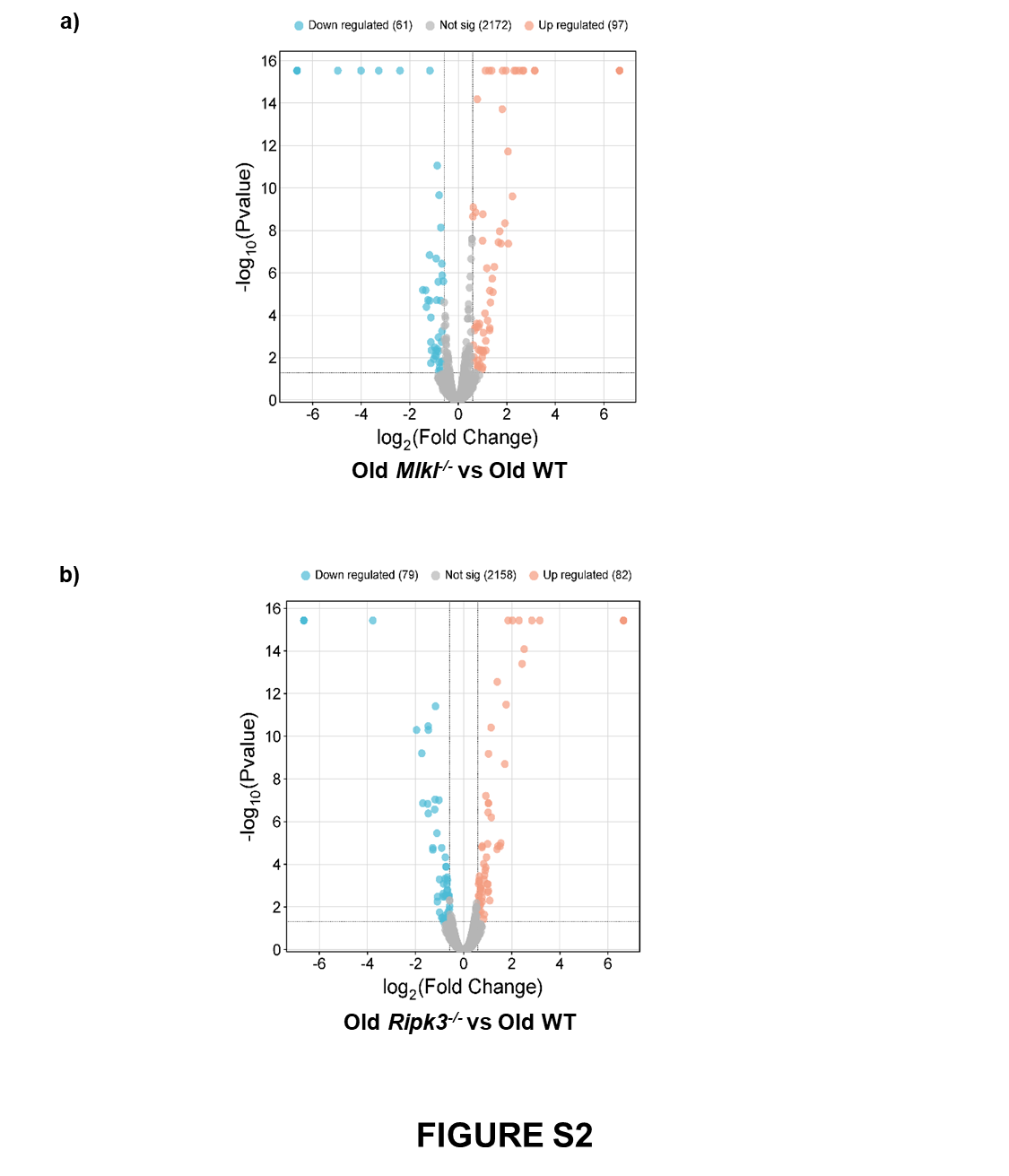
**

**Fig. S2** Volcano plot analysis of significantly modulated proteins. (a) old *Mlkl^-/-^* mice and (b) old *Ripk3^-/-^* mice when compared to old WT. Significant proteins represented with a fold change of at least 1.5 with an adjusted p-value > 0.05. N=5 per group


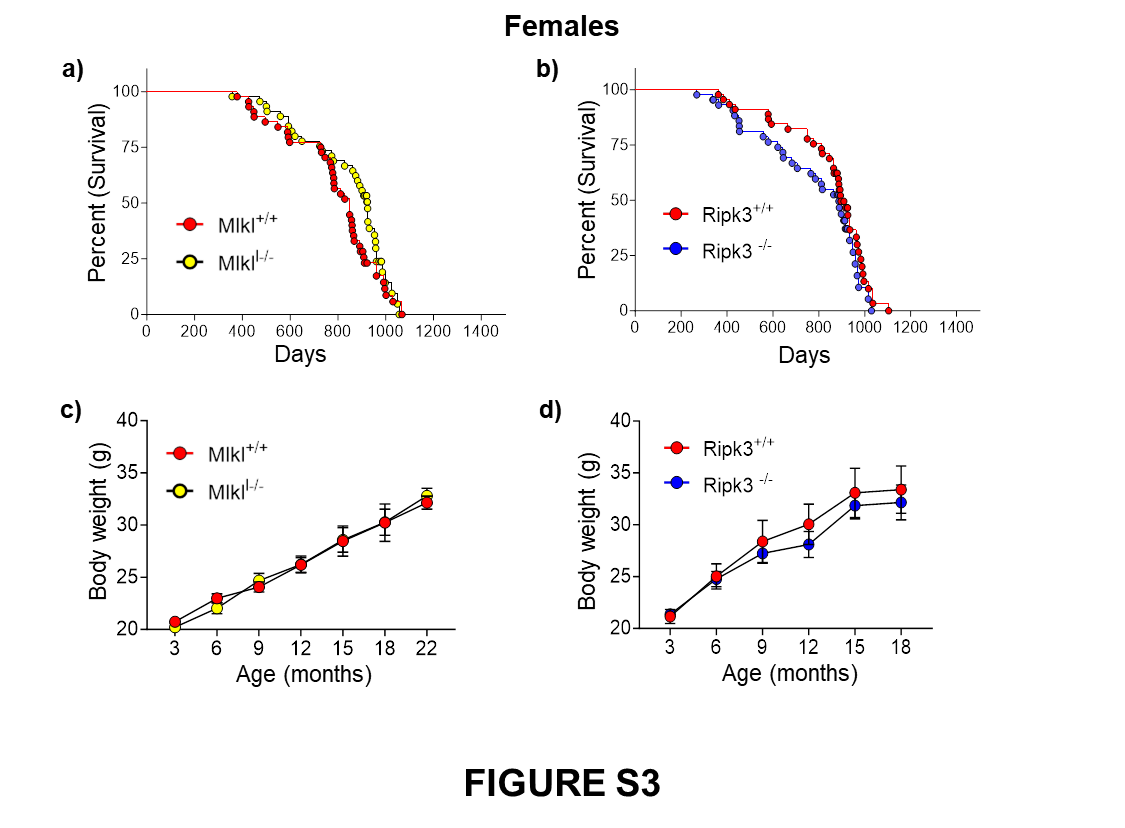


**Fig. S3** Effect of absence of *Ripk3 or Mlkl* on survival and body weight of female mice. Kaplan-Meier survival curve for a) *Mlkl^+/+^, Mlkl^-/-^* (on the left) or (b) *Ripk3^+/+^, Ripk3^-/-^* (on the right). Average body weight changes of (c) *Mlkl^+/+^* , or *Mlkl^-/-^* and (d) *Ripk3^+/+^ , Ripk3^-/-^* mice. a, b: n= 44 *Mlkl^+/+^*, n=45 *Mlkl^-/-^ ,* n=45 *Ripk3^+/+^*, n=44 *Ripk3^-/-^*. c, d: n=10/ group. Data are expressed as mean ± SEM and analyzed using a two-way ANOVA. ANOVA summary (F value, p value): (c) row factor (time points) 42.53, <0.0001 column factor (genotype) 0.00038, 0.995; (d) row factor (time points) 18.61, <0.0001 column factor (genotype) 1.21, 0.273

**
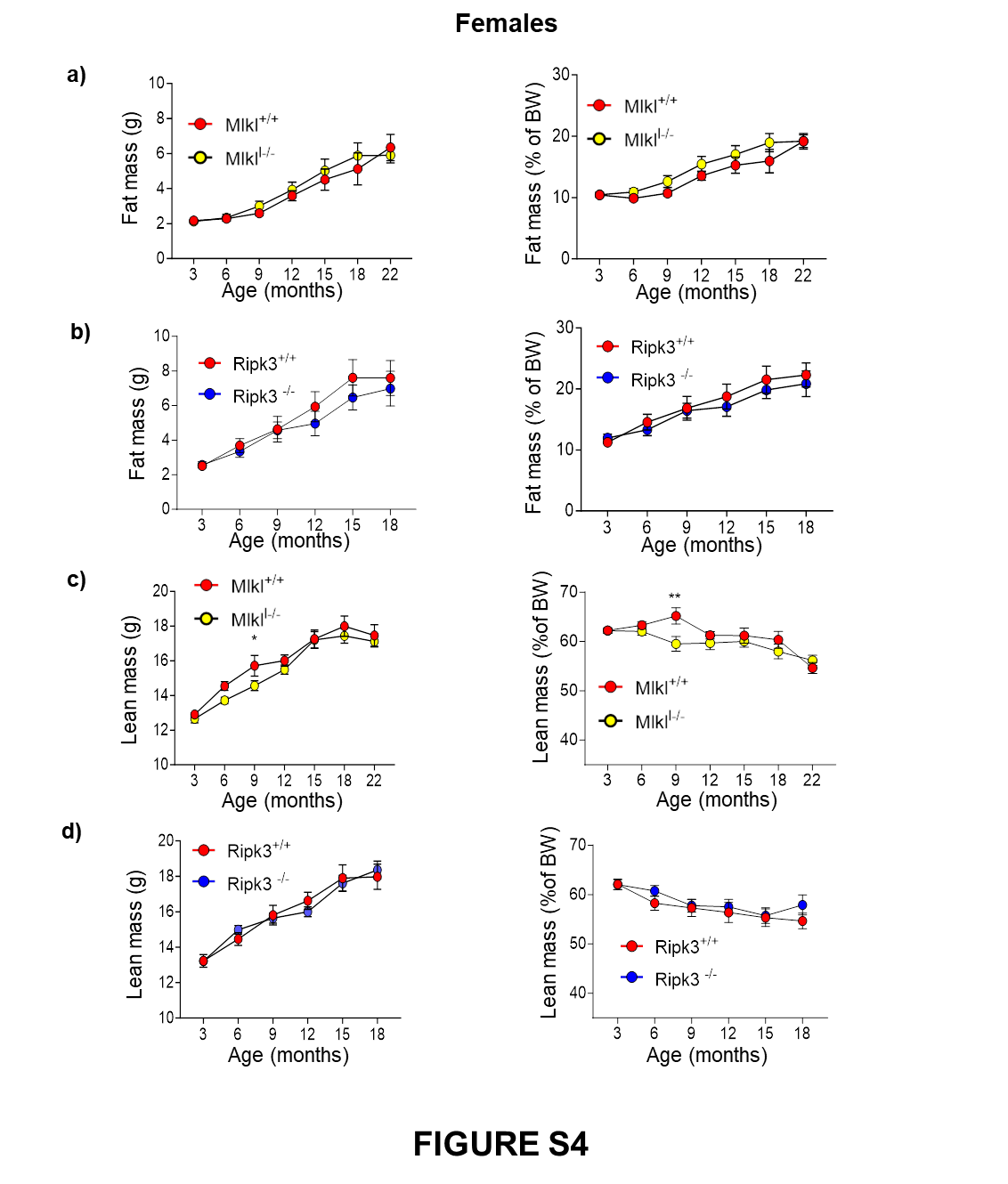
**

**Fig. S4** Effect of absence of *Ripk3 or Mlkl* on body composition of female mice. Gross fat mass (in grams) (on the left) and percentage of fat mass normalized to total body weight (on the right) of (a) *Mlkl^+/+^*, *Mlkl^-/-^* mice and (b) *Ripk3^+/+^, Ripk3^-/-^* mice. Gross lean mass (in grams) (on the left) and percentage of lean mass normalized to total body weight (on the right) of (c) *Mlkl^+/+^*, *Mlkl^-/-^* mice and (d *Ripk3^+/+^, Ripk3^-/-^* mice. n=10/group. Data are expressed as mean ± SEM and analyzed using a two-way ANOVA. * p< 0.05, ** p< 0.005, *** p<0.0005. ANOVA summary (F value, p value): (a, left) row factor (time points) 21.78, <0.0001 column factor (genotype) 0.78, 0.38; (a, right) row factor (time points) 20.85, <0.0001 column factor (genotype) 5.56, 0.02; (b, left) row factor (time points) 15.13, <0.0001 column factor (genotype) 1.65, 0.20; (b, right) row factor (time points) 12.16, <0.0001 column factor (genotype) 1.11, 0.295; (c, left) row factor (time points) 42.2, <0.0001 column factor (genotype) 6.09, 0.015; (c, right) row factor (time points) 8.5, <0.0001 column factor (genotype) 5.27, 0.023; (d, left) row factor (time points) 34.67, <0.0001 column factor (genotype) 0.015, 0.90; (d, right) row factor (time points) 4.92, 0.005 column factor (genotype) 2.06, 0.154


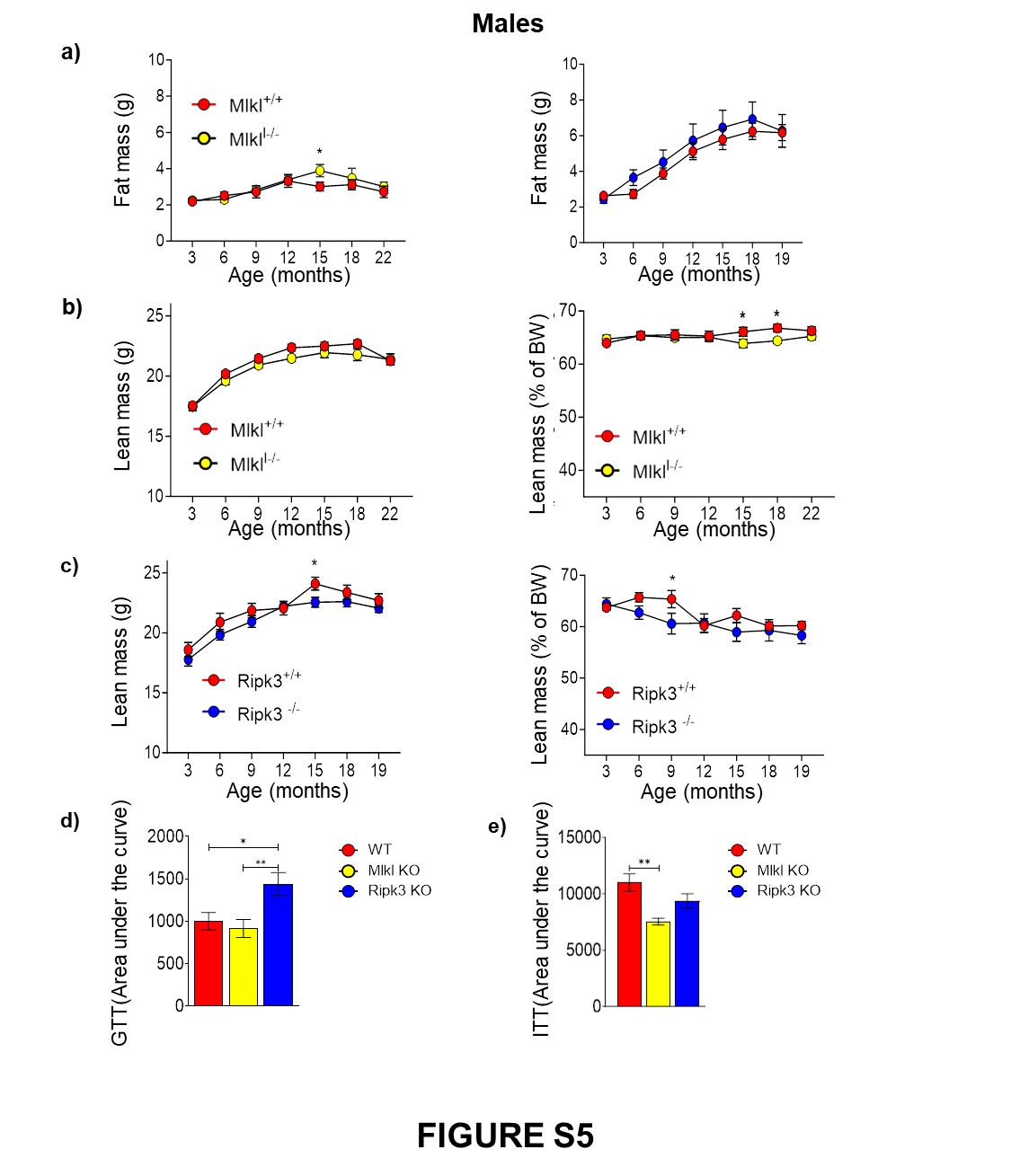


**Fig. S5** Effect of absence of *Ripk3 or Mlkl* on body composition of male mice. (a) Gross fat mass (in grams) *Mlkl^+/+^*, *Mlkl^-/-^* mice (on the left) and *Ripk3^+/+^, Ripk3^-/-^* mice (on the right). (b) Gross

lean mass (in grams) (on the left) and percentage of lean mass normalized to total body weight (on the right) of *Mlkl^+/+^*, *Mlkl^-/-^* mice (c) Gross lean mass (in grams) (on the left) and percentage

of lean mass normalized to total body weight (on the right) of *Ripk3^+/+^, Ripk3^-/-^* mice. Area under the curve for (d) GTT represented in Figure 6g and (e) ITT represented in Figure 6h. ANOVA summary (F value, p value): (a, left) row factor (time points) 6.04, <0.0001 column factor (genotype) 2.17, 0.143; (a, right) row factor (time points) 13.72, <0.0001 column factor (genotype) 2.2, 0.140; (b, left) row factor (time points) 55.22, <0.0001 column factor (genotype) 7.752, 0.006; (b, right) row factor (time points) 0.73, 0.63 column factor (genotype) 3.98, 0.048; (c, left) row factor (time points) 22.66, <0.0001 column factor (genotype) 7.750, 0.0063; (c, right) row factor (time points) 3.87, 0.0015 column factor (genotype) 4.87, 0.029; (d) 5.38, 0.012; (e) 6.87, 0.006

**TABLE S1** List of proteins identified by label-free quantitative proteomic analysis of liver tissues from young WT, old WT, old *Mlkl^-/^*^-^, and old *Ripk3^-/-^* mice.

| **Proteins commonly upregulated in old *Mlkl^-/-^* and old *Ripk3^-/-^*** | **Proteins commonly downregulated in old *Mlkl^-/-^* and old *Ripk3^-/-^*** |
| --- | --- |
| UBXN1  TSNAX  STX8  SF3B4  RPL37  PTRHD1  PCNA  MTMR3  LRG1  IQGAP2  IPO9  FCER1G  FAM114A1  ERLIN2  CSRP2  CD74  CAMK2A  AFDN  ACNAT2  PDGFC  IGSF9B  MUP17  SLCO2B1  ACOT3  ABHD12  NUCKS1  ABLIM1  MRPL50  RNF185  SELENBP2  TOMM5  DIAPH1 | PKLR  H1-5  UGT1A9  CD81  NAPA  S100A11  IFGGD1  UBE2L6  NT5C  U2AF2  ARL1  JPT2  ZC3H15  TBC1D5  SHFL  LYPLA2  ITPRID2  ERBIN  DSC2  DHRS3  DDX21 |

**TABLE S2** Sequences of quantitative PCR primers used in the study.

| **Gene** | **Forward sequence** | **Reverse sequence** |
| --- | --- | --- |
| Acta2 | 5’-CTGACAGAGGCACCACTGAA-3’ | 5’-CATCTCCAGAGTCCAGCACA-3’ |
| Arg1 | 5’-CTCCAAGCCAAAGTCCTTAGAG-3’ | 5’-AGGAGCTGTCATTAGGGACATC-3’ |
| β- actin | 5′-ATGGATGACGATATCGCTG-3′ | 5′-GTTGGTAACAATGCCATGTTC-3′ |
| β-microglobulin | 5′-CACTGACCGGCCTGTATGC-3′ | 5′-GGGTGGCGTGAGTATACTTGAAT-3′ |
| Cdkn2a (p16^Ink4a^) | 5’-CCCAACGCCCCGAACT-3’ | 5’-GCAGAAGAGCTGCTACGTGAA-3’ |
| Cdkn1a (p21^Cip1^) | 5’-GTCAGGCTGGTCTGCCTCCG-3’ | 5’-CGGTCCCGTGGACAGTGAGCAG-3’ |
| Col1α1 | 5’-GCTCCTCTTAGGGGCCACT-3’ | 5’-CCACGTCTCACCATTGGGG-3’ |
| CD11c | 5’-CTGGATAGCCTTTCTTCTGCTG-3’ | 5’-GCACACTGTGTCCGAACTC-3’ |
| CD68 | 5’-CCACAGGCAGCACAGTGGAC-3’ | 5’-TCCACAGCAGAAGCTTTGGCCC-3’ |
| CD86 | 5’-ACGATGGACCCCAGATGCACCA-3’ | 5’-GCGTCTCCACGGAAACAGCA-3’ |
| CXCL2 | 5’-CCTGGTTCAGAAAATCATCCA-3’ | 5’-CTTCCGTTGAGGGACAGC-3’ |
| Fizz1 | 5’-CCAATCCAGCTAACTATCCCTCC-3’ | 5’-CCAGTCAACGAGTAAGCACAG-3’ |
| HPRT | 5’-CTGGTGAAAAGGACCTCTCG-3’ | 5’-TGAAGTACTCATTATAGTCAAGGGCA-3’ |
| IL1β | 5’-AGGTCAAAGGTTTGGAAGCA-3’ | 5’-TGAAGCAGCTATGGCAACTG-3’ |
| IL-6 | 5’-TGGTACTCCAGAAGACCAGAGG-3’ | 5’-AACGATGATGCACTTGCAGA-3’ |
| MCP1 | 5’-TTAAAAACCTGGATCGGAACCAA-3’ | 5’-GCATTAGCTTCAGATTTACGGGT-3’ |
| MLKL | 5′-CTGAGGGAACTGCTGGATAGAG-3′ | 5′-CGAGGAAACTGGAGCTGCTGAT-3′ |
| MMP3 | 5’-GTTGGAGAACATGGAGACTTTGT-3’ | 5’-CAAGTTCATGAGCAGCAACCA-3’ |
| MMP12 | 5’-TGCACTCTGCTGAAAGGAGTCT-3’ | 5’-GTCATTGGAATTCTGTCCTTTCCA-3’ |
| P19 | 5'-GGGTCGCAGGTTCTTGGTC-3' | 5'-AATCTGCACCGTAGTTGAGCA-3' |
| RIPK3 | 5′-GAAGACACGGCACTCCTTGGTA-3′ | 5′-CTTGAGGCAGTAGTTCTTGGTGG-3′ |
| TGFβ | 5’-ACCATGCCAACTTCTGTCTGGGAC-3’ | 5’-ACAACTGCTCCACCTTGGGCTTG-3’ |
| TNFα | 5’-CACAGAAAGCATGATCCGCGACGT-3’ | 5’- CGGCAGAGAGGAGGTTGACTTTCT-3’ |
